# Backbone Steric Constraints Underlie High Passive Membrane Permeability of *N*‐Alkyl Peptides

**DOI:** 10.1002/anie.3948218

**Published:** 2026-08-06

**Authors:** Ayumi Inayoshi, Mariko Akiba, Marin Yokomine, Masatake Sugita, Koji Umezawa, Yuto Hirota, Yutaka Akiyama, Jumpei Morimoto, Shinsuke Sando

**Affiliations:** ^1^ Department of Chemistry and Biotechnology Graduate School of Engineering The University of Tokyo Bunkyo‐ku Tokyo Japan; ^2^ Present address: Department of Chemistry, Graduate School of Science Kyoto University Sakyo‐ku Kyoto Japan; ^3^ Department of Computer Science School of Computing Institute of Science Tokyo Meguro Tokyo Japan; ^4^ Middle Molecule IT‐based Drug Discovery Laboratory (MIDL) Institute of Science Tokyo Meguro Tokyo Japan; ^5^ Department of Biomedical Engineering Graduate School of Science and Technology Shinshu University Kami‐ina Nagano Japan; ^6^ Institute for Biomedical Sciences Interdisciplinary Cluster for Cutting Edge Research Shinshu University Matsumoto Nagano Japan; ^7^ Department of Bioengineering Graduate School of Engineering The University of Tokyo Bunkyo‐ku Tokyo Japan

**Keywords:** molecular dynamics simulations, *N*‐alkyl peptides, passive membrane permeability, peptides, steric constraints

## Abstract

*N*‐Alkyl peptides have emerged as promising drug modalities, yet the structural determinants governing passive membrane permeability beyond amide hydrogen removal remain poorly understood. Here, we show that sterically constrained *N*‐alkyl peptide backbones, generated by dual substitution at the amide nitrogen and the α‐carbon, play a critical role in promoting passive membrane permeability. By directly comparing *N*‐alkyl peptides with oligo(*N*‐alkyl glycines) lacking *C*α‐substituents, we isolated the backbone steric effects independently of amide hydrogen removal. *N*‐Alkyl peptides bearing an *N*/*C*α‐dually substituted backbone architecture consistently exhibited enhanced permeability across a broad range of lipophilicity and diverse sequences. Molecular dynamics simulations revealed two cooperative mechanisms: conformational restriction that favors less hydrated states and steric limitation of backbone hydration by β‐carbons during membrane permeation. Together, these findings uncover a previously unrecognized structural basis for the high passive membrane permeability of *N*‐alkyl peptides.

## Graphical abstract

Beyond amide hydrogen removal, steric constraints encoded in *N*/*C*α‐dually substituted backbones are uncovered as a key determinant of passive membrane permeability in *N*‐alkyl peptides. These constraints restrict conformational freedom and sterically limit backbone hydration during membrane permeation.

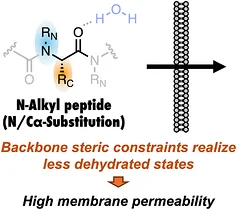

## 1 Introduction

*N*‐Alkyl peptides have attracted considerable attention as medium‐sized drug modalities that are orally bioavailable and capable of targeting intracellular biomolecules owing to their high passive membrane permeability [1, 2, 3]. For example, the naturally occurring cyclic peptide cyclosporin A (CsA) is a representative *N*‐alkyl peptide in which many backbone amide nitrogens are *N*‐methylated (Figure 1a); it passively permeates cell membranes and exerts potent biological activity through interactions with intracellular calcineurin and cyclophilin A [4, 5]. When an amide bond is *N*‐alkylated, the amide hydrogen that serves as a hydrogen‐bond donor is removed, thereby reducing the desolvation energy required for membrane permeation and enhancing the passive membrane permeability (Figure 1b) [6, 7, 8, 9, 10, 11]. Inspired by the prevalence of *N*‐alkyl amide structures in naturally occurring membrane‐permeable peptides, substantial research efforts have focused on developing synthetic *N*‐alkyl peptides that target intracellular proteins [12, 13, 14].

**FIGURE 1:**
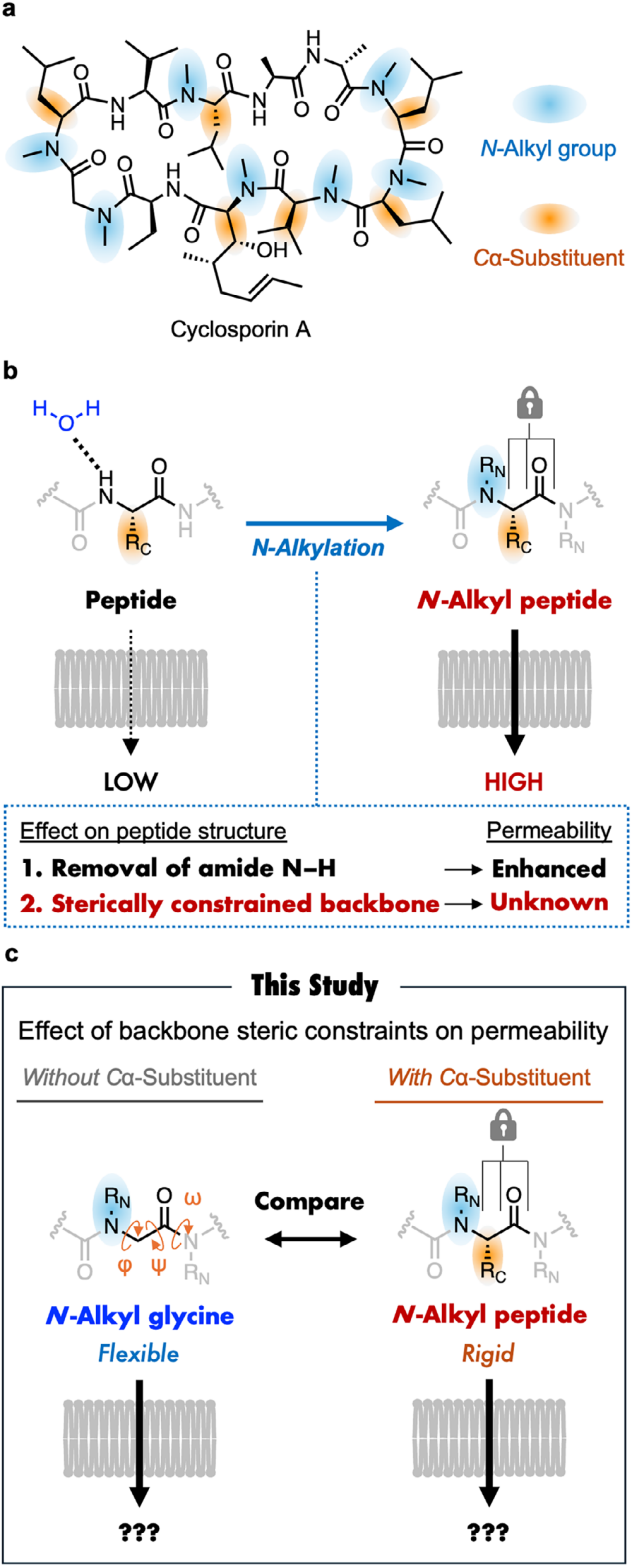
(a) A representative *N*‐alkyl peptide, cyclosporin A (CsA). *N*‐Alkyl groups and *C*α‐substituents corresponding to amino acid side chains are highlighted in blue and orange, respectively. (b) Effects of *N*‐alkylation on peptide structure and its influence on membrane permeability. (c) Schematic comparison of *N*‐alkyl glycine (left) and *N*‐alkyl peptide (*N*/*C*α‐dually substituted) (right) used in this study to evaluate the effect of backbone steric constraints on membrane permeability.

An important structural feature of *N*‐alkyl peptides is their sterically constrained backbone architecture. Cooperative steric repulsion between *N*‐alkyl groups on the amide nitrogen and *C*α substituents corresponding to amino‐acid side chains markedly restricts the conformational freedom of the peptide backbone (Figure 1b, right) [15, 16, 17]. Specifically, the amide bond equilibrium is biased toward the *trans* configuration and, through pseudo‐1,3‐allylic strain, the rotational freedom of the backbone dihedral angles φ and ψ is reduced (Figure 1b, right). Such conformational restriction enhances binding affinity and selectivity toward target proteins [16, 18, 19, 20, 21], thereby increasing the utility of *N*‐alkyl peptides in drug discovery. In contrast, how backbone steric constraints influence passive membrane permeability remains poorly understood (Figure 1b, bottom).

Even among peptides bearing identical *N*‐alkyl groups, oligomers of *N*‐alkyl glycines, often referred to as oligo‐NSG (*N*‐substituted glycines) or peptoids [22], lack substituents at the *C*α position and therefore do not experience local steric repulsion (Figure 1c, left). Consequently, their backbone dihedral angles (φ, ψ, and ω) retain high rotational freedom compared with *N*‐alkyl peptides bearing an *N*/*C*α‐dually substituted backbone architecture.

Based on these considerations, we reasoned that comparing *N*‐alkyl peptides (here defined as *N*/*C*α‐dually substituted peptides; Figure 1c, right) with oligo‐NSG lacking *C*α substituents (Figure 1c, left) would enable direct evaluation of backbone steric effects on passive membrane permeability. Because both classes of molecules lack backbone amide hydrogens, this comparison allows evaluation of the contribution of backbone steric constraints independently of hydrogen‐bond donor removal.

Through this comparative analysis, we demonstrate that an *N*/*C*α‐dually substituted backbone architecture of *N*‐alkyl peptides promotes passive membrane permeability by restricting conformational freedom and sterically limiting backbone hydration.

## 2 Results and Discussions

### 2.1 Effect of the Backbone Architecture on the Membrane Permeability of *N*‐Alkyl Peptides

To evaluate the effect of backbone steric constraints on the membrane permeability of *N*‐alkyl peptides, we designed oligo‐NSG and an oligomer of *N*‐alkyl alanines bearing a methyl group at the *C*α position (oligo‐NSA) (Figure 2a). When oligo‐NSG and oligo‐NSA of identical chain length bearing the same *N*‐substituents are compared, oligo‐NSA inevitably exhibits higher calculated lipophilicity (ALogP) because of the additional methyl group at the α‐carbon. To enable comparison between molecules with comparable lipophilicity, we therefore designed **NSG1** and **NSA2** with different *N*‐substituents such that their ALogP values would be similar. Experimentally determined LogP values, measured by a chromatographic method [23], were 4.8 and 4.6 for **NSG1** and **NSA2**, respectively, confirming that lipophilicity was approximately matched. Thus, **NSG1** and **NSA2** provide a system in which backbone steric effects can be evaluated independently of lipophilicity.

**FIGURE 2:**
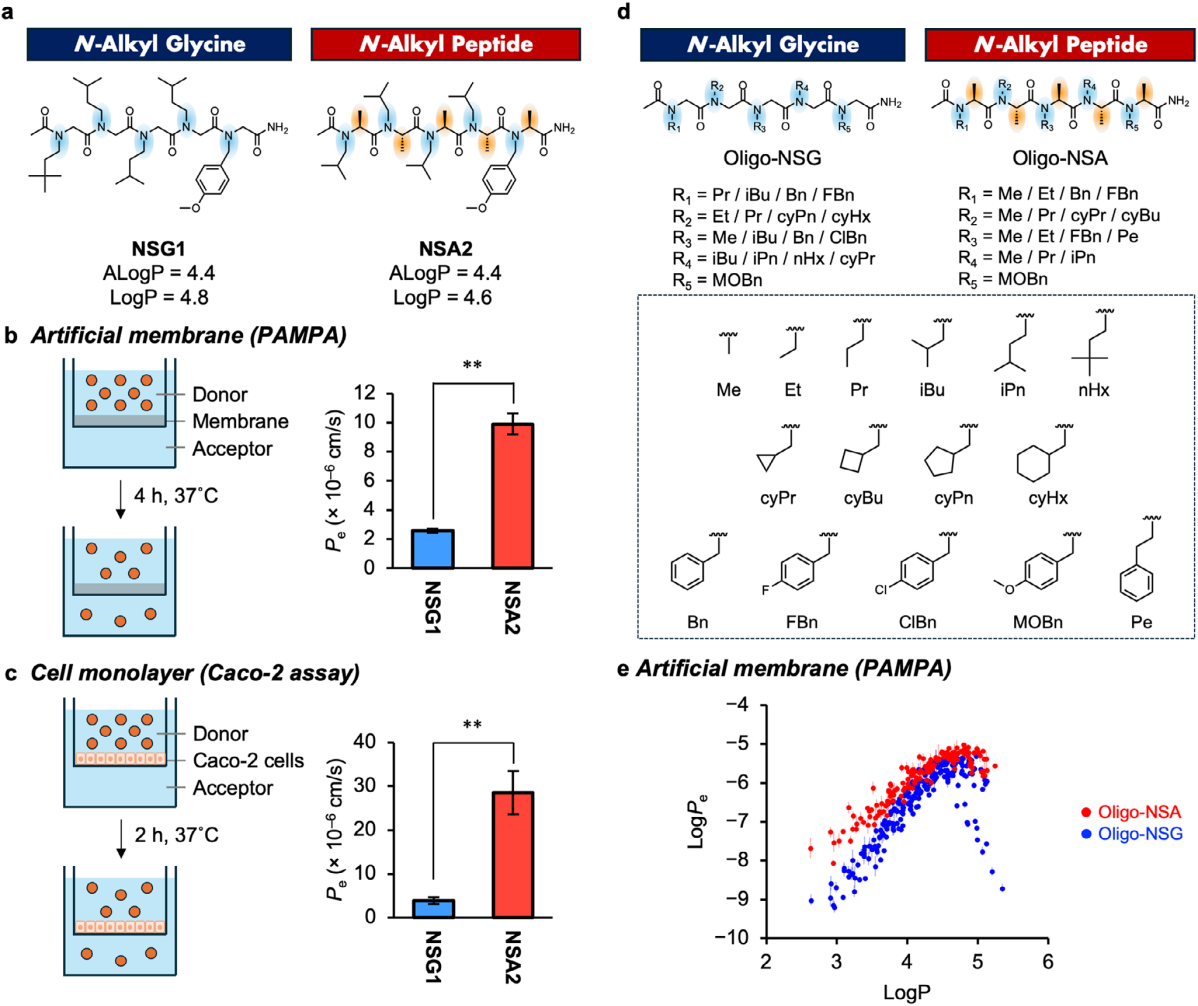
(a) Chemical structures of **NSG1** and **NSA2**. ALogP and experimentally determined LogP values are shown below the structures. (b) (Left) Schematic illustration of PAMPA. (Right) Permeability coefficients (*P*_e_) of **NSG1** and **NSA2** determined by PAMPA. *P*_e_ values represent the mean ± standard deviation of triplicate measurements. Statistical significance was evaluated using Tukey's test (**p < 0.01). (c) (Left) Schematic illustration of Caco‐2 assay. (Right) *P*_e_ determined by Caco‐2 assay (apical to basolateral transport). Permeability was measured in the presence of verapamil (100 µM). *P*_e_ values represent the mean ± standard deviation of eight technical replicates for **NSG1** and four technical replicates for **NSA2**. Statistical significance was evaluated using Tukey's test (**p < 0.01). (d) Structures of the oligo‐NSG and oligo‐NSA libraries. Top: general structures and side‐chain repertoire. Bottom: individual *N*‐substituent structures. (e) Dot plots of Log*P*_e_ versus LogP for the oligo‐NSG (blue) and oligo‐NSA (red) libraries.

Membrane permeability of **NSG1** and **NSA2** was evaluated using the parallel artificial membrane permeability assay (PAMPA). **NSA2** exhibited significantly higher membrane permeability than **NSG1**, indicating that the presence of *C*α‐substituents enhances passive membrane permeability in *N*‐alkyl peptides (Figure 2b).

We used PAMPA because this assay enables evaluation of passive membrane permeability while minimizing the influence of active transport, efflux, metabolism, and other cell‐dependent processes. To examine whether the permeability trend observed in PAMPA is also reflected in a cellular membrane context, we performed a Caco‐2 assay. Consistent with the PAMPA results, **NSA2** displayed higher membrane permeability than **NSG1** in the cell‐based assay (Figure 2c).

### 2.2 Evaluation of Membrane Permeability Using a Combinatorial Library

To examine whether the backbone effect is general across diverse sequences, we constructed a combinatorial library in which *N*‐substituents were systematically varied to cover a broad range of lipophilicity (Figure 2d). The library was synthesized using a split‐and‐pool strategy, and membrane permeability was evaluated by PAMPA. In total, 230 oligo‐NSGs and 150 oligo‐NSAs were analyzed. As shown in Figure S1, oligo‐NSA and oligo‐NSG cover largely overlapping ranges of calculated as well as experimentally determined lipophilicity, enabling comparison of membrane permeability with lipophilicity‐matched conditions.

Across a wide range of lipophilicity, oligo‐NSA consistently exhibited higher membrane permeability than oligo‐NSG when compounds with comparable LogP values were compared (Figures 2e and S2). Both series showed an approximately parabolic relationship between LogP and Log*P*_e_, consistent with trends previously reported for cyclic peptides [24, 25]. Notably, the difference in permeability between oligo‐NSA and oligo‐NSG was most pronounced among sequences with relatively low LogP values, whereas the difference became smaller for highly lipophilic sequences (Figure S2). This trend likely arises because, as *N*‐substituents become larger and more lipophilic, side‐chain contributions increasingly dominate membrane permeability, thereby diminishing the relative impact of backbone architecture.

Taken together, the library‐based analysis demonstrates that backbone steric constraints promote passive membrane permeability of *N*‐alkyl peptides across a broad lipophilicity range and diverse sequence space.

### 2.3 Comparison of Membrane Permeability Among *N*‐Alkyl Peptides With Different Stable Conformations

**NSA2**, evaluated in the experiments described above, is expected to adopt an extended, linear backbone conformation as a result of local steric repulsion, based on previous structural studies [16]. Therefore, it is possible that the high membrane permeability of this peptide arises not from the backbone steric constraints per se but from its linear molecular shape.

To examine whether molecular shape is a dominant determinant of passive membrane permeability, we prepared *N*‐alkyl peptides exhibiting a range of molecular shapes by combining l‐ and d‐amino acids and compared their membrane permeability with that of the conformationally flexible oligo‐NSG. Previous studies have shown that *N*‐alkyl peptides adopt distinct stable conformations depending on the stereochemical sequence of l‐ and d‐amino acids [26]. Accordingly, we measured the membrane permeability of 16 stereoisomers generated by varying the L/D configurations of each residue in **NSA2** using PAMPA and compared the results with those of **NSG1** (Figure 3a,b). Principal moment of inertia (PMI) analysis revealed that these stereoisomers occupy distinct regions of shape space, indicating that they adopt diverse molecular geometries. As shown in Figure 3a (bottom), variants with different stereochemical sequences, such as LLLLL (**NSA2**) and DDLDL (**NSA3**), exhibit markedly different molecular shapes. If molecular shape were the dominant determinant of permeability, a correlation between PMI distribution and membrane permeability would be expected; however, such a trend was not observed.

**FIGURE 3:**
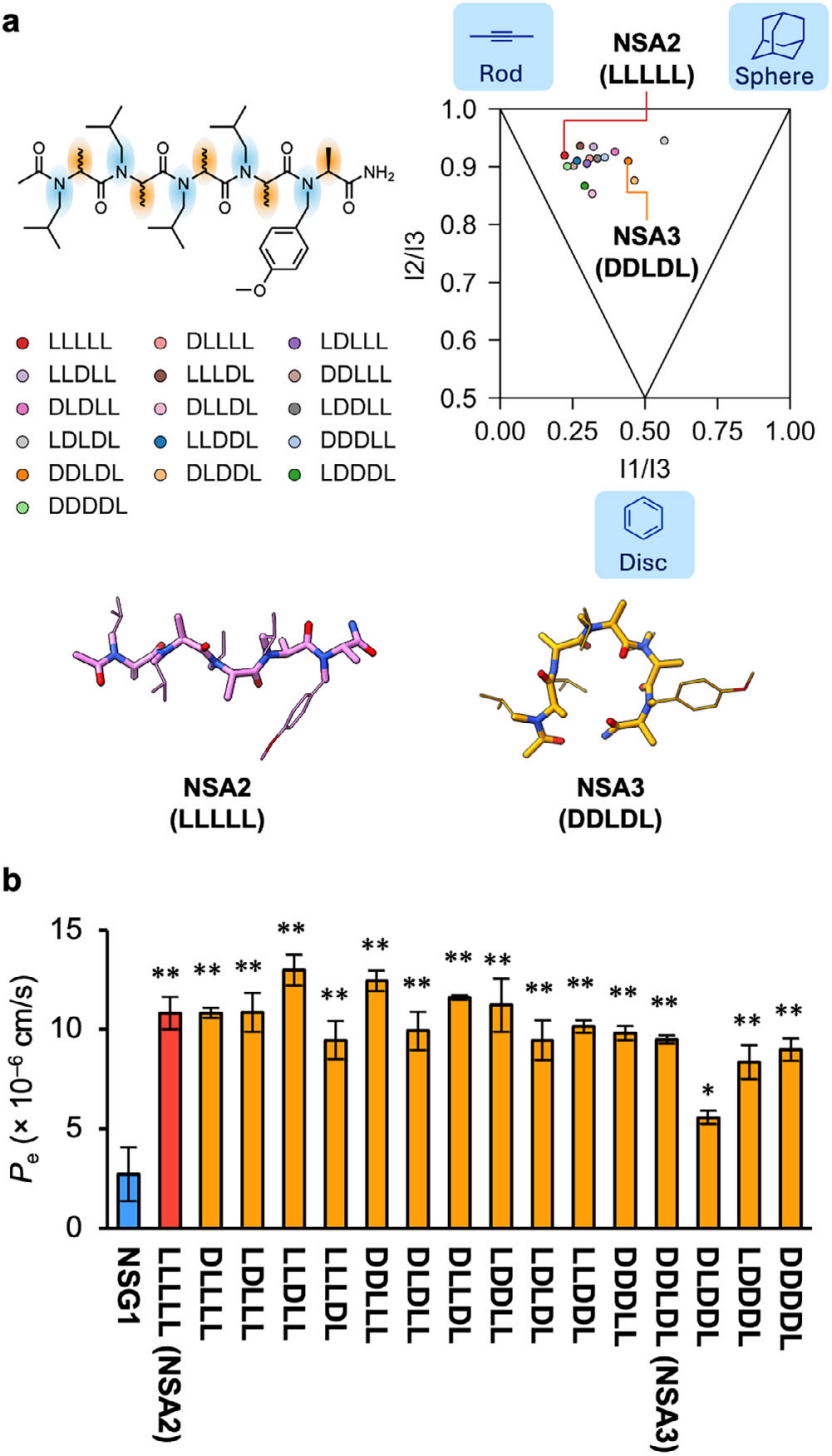
(a) PMI plot of heterochiral oligo‐NSA variants. Stable conformations of **NSA2** and **NSA3** are shown below. (b) *P*_e_ determined by PAMPA. *P*_e_ values represent the mean ± standard deviation of nine technical replicates for **NSG1** and three technical replicates for oligo‐NSAs. Statistical significance relative to **NSG1** is shown. Statistical analysis was performed using Tukey's test (**p < 0.01, *p < 0.05).

PAMPA measurements showed that all oligo‐NSA variants exhibited higher membrane permeability than oligo‐NSG, despite modest variations among stereoisomers (Figure 3b). To further evaluate whether this trend holds in living cells, we performed a Caco‐2 assay using **NSG1** and two oligo‐NSA variants (**NSA2** and **NSA3**) that occupy distant positions on the PMI plot and therefore possess distinct molecular shapes (Figure 3a, bottom). Consistent with the PAMPA results, both oligo‐NSA variants displayed higher membrane permeability than oligo‐NSG in the cellular assay (Figure S3).

Together, these findings indicate that high membrane permeability cannot be uniquely attributed to a specific molecular shape among the examined oligo‐NSA stereoisomers. Instead, they suggest that the sterically constrained backbone architecture bearing *N*/*C*α‐dual substitutions is a key contributor to passive membrane permeation in *N*‐alkyl peptides. We note, however, that this conclusion does not rule out the possibility that molecular shape may influence permeability in other peptide or peptidomimetic backbone architectures.

### 2.4 Molecular Dynamics Simulation of the Membrane Permeation Process of *N*‐Alkyl Peptides

To understand why sterically constrained backbone structures in *N*‐alkyl peptides lead to pronounced differences in membrane permeability, we performed structural analyses using molecular dynamics (MD) simulations. Simulations were conducted for **NSG1** and **NSA2** in a system containing water and a lipid bilayer. The membrane permeation process was inferred by analyzing ensembles of conformations sampled using two‐dimensional replica‐exchange simulations involving 224 replicas that differed in both temperature and distance from the membrane center (Figure 4a) [27, 28, 29].

**FIGURE 4:**
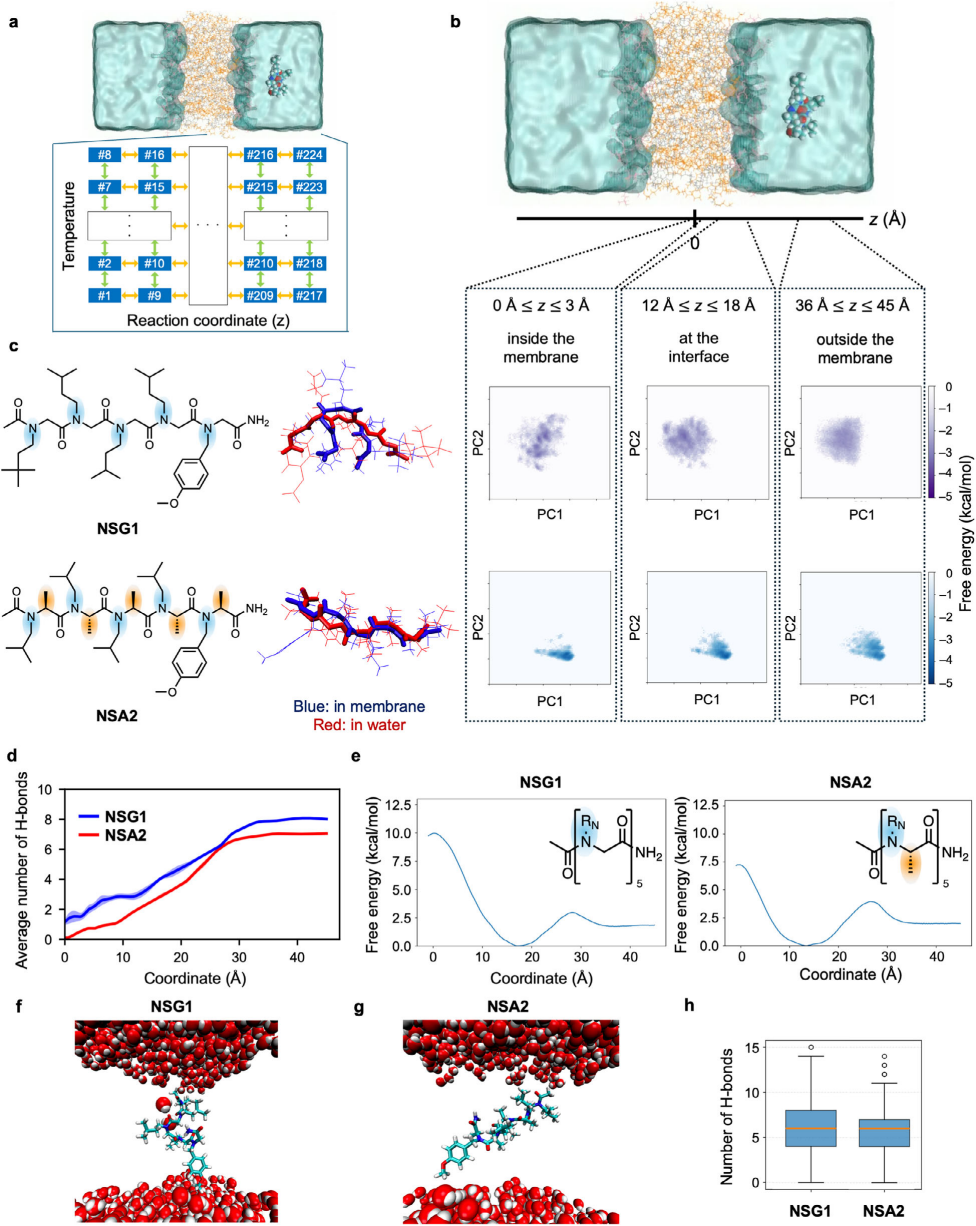
(a) Schematic illustration of the MD simulation setup. (b) Principal component analysis (PCA) of sampled conformations of **NSG1** (purple) and **NSA2** (blue). (c) (Left) Chemical structures of **NSG1** and **NSA2**. (Right) Representative conformations in the membrane (blue) and in water (red). (d) Average number of hydrogen bonds formed between the peptides and water molecules at 300 K for **NSG1** (blue) and **NSA2** (red). Shaded regions represent standard errors of the mean calculated over 100 ns intervals. (e) Free‐energy profiles along the coordinate for **NSG1** (left) and **NSA2** (right). (f and g) Representative snapshots of **NSG1** and **NSA2** at the membrane center; lipid molecules are omitted for clarity. (h) Box plot showing the distribution of hydrogen bonds formed with water molecules across all temperature trajectories. Note that the convergence of the hydrogen‐bond profiles was confirmed for all replicas. As an example, the hydrogen‐bond profiles of **NSA2** for all REST replicas within the REUS replica located farthest from the membrane, along the reaction coordinate, are shown in Figure S4.

The MD simulations suggested that **NSG1**, which lacks *C*α‐substituents, adopts a wide range of conformations both in aqueous solution and within the lipid membrane (Figure 4b,c, purple PCA plot and top, respectively). The average number of hydrogen bonds formed between **NSG1** and water molecules (Figure 4d, blue) was consistently higher than that observed for **NSA2** (Figure 4d, red) across all regions, from bulk water to the membrane center. Notably, even within the membrane interior, the number of hydrogen bonds rarely reached zero, suggesting that **NSG1** often remains partially hydrated and does not fully dehydrate after entering the membrane. The free‐energy difference between bulk water and the membrane center was substantially larger for **NSG1** (Figure 4e), likely reflecting destabilization of the system when the peptide resides within the membrane due to incomplete desolvation.

In contrast, **NSA2**, which is an *N*/*C*α‐dually substituted peptide, adopted similar conformations across the entire trajectory from bulk water to the membrane center (Figure 4b, blue PCA plot). In both aqueous and membrane environments, representative structures corresponding to free‐energy minima projected onto PC1 and PC2 exhibited extended conformations (Figure 4c, bottom), consistent with previous structural analyses of oligo‐NSA [19, 26]. The number of hydrogen bonds formed between **NSA2** and water molecules was lower than that of **NSG1** at all positions and decreased to nearly zero within the membrane interior (Figure 4d, red). Compared with **NSG1**, **NSA2** displayed a smaller free‐energy difference between bulk water and the membrane center (Figure 4e), suggesting that its backbone architecture favors less hydrated states and reduces the energetic cost of crossing the membrane center.

Based on these differences in hydration behavior and conformational properties, two structural factors likely account for the high membrane permeability of *N*‐alkyl peptides bearing an *N*/*C*α‐dually substituted backbone architecture such as **NSA2** (Figure 5).

**FIGURE 5:**
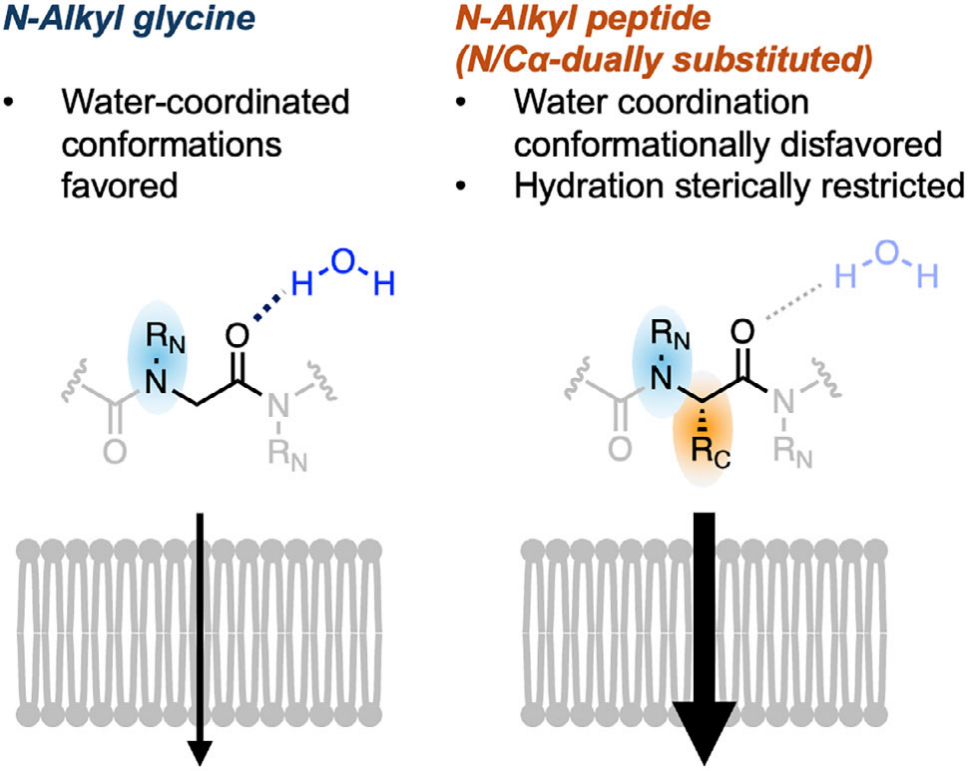
Proposed mechanistic model illustrating how sterically constrained backbone architectures of *N*‐alkyl peptides enhance passive membrane permeability.

First, the combined presence of *N*‐alkyl groups and *C*α‐substituents restricts overall conformational freedom. As a result, the formation of stable peptide–water complexes becomes less favorable, promoting less hydrated states as the peptide approaches the membrane center. Inspection of representative structures revealed that **NSG1** frequently adopts conformations in which a water molecule is coordinated by two carbonyl oxygen atoms, allowing water to accompany the peptide into the membrane (Figure 4f). In contrast, **NSA2** is almost completely dehydrated at the membrane center (Figure 4g).

Second, β‐carbons sterically hinder the approach of water molecules to backbone carbonyl oxygen atoms, intrinsically reducing backbone hydration. Indeed, the maximum number of hydrogen bonds potentially formed between the peptides and water observed in bulk water (*z* = 36–45 Å) was lower for **NSA2** than for **NSG1** (Figure 4h). These observations suggest that **NSA2** provides a smaller accessible spatial region for water molecules to approach polar backbone atoms. Supporting this interpretation, removal of methyl groups from a representative **NSA2** conformation obtained from the MD simulations increased the solvent‐accessible surface area of carbonyl oxygen atoms (Figure S5).

Taken together, these findings indicate that sterically constrained backbone architectures promote passive membrane permeability by favoring less hydrated conformational states during membrane permeation.

We note that the experimental results of the present study are primarily limited to oligo‐NSAs bearing *C*α‐methyl substituents. Larger *C*α‐substituents may further modulate backbone hydration and membrane permeability by altering steric shielding around the backbone carbonyl groups. We therefore examined this possibility computationally. Specifically, we performed 500‐ns REST simulations in water for **NSA2** analogs bearing ethyl or isopropyl substituents at the *C*α positions. Compared with **NSA2** bearing *C*α‐methyl substituents, the ethyl‐substituted analog formed fewer hydrogen bonds with water, whereas the isopropyl‐substituted analog showed no further decrease in hydrogen bonding relative to the ethyl‐substituted analog (Figure S6). These results suggest that larger *C*α‐substituents can more effectively shield backbone carbonyl oxygens from water, although this effect may reach a plateau beyond a certain substituent size. Experimental investigation of the membrane permeability of *N*‐alkyl peptides bearing larger *C*α‐substituents will be an interesting direction for future studies.

### 2.5 Effects of Backbone Steric Constraints on Membrane Permeability of Bioactive *N*‐Alkyl Peptides

Finally, we examined whether backbone steric constraints also influence the membrane permeability of bioactive *N*‐alkyl peptides (Figure 6a,b).

**FIGURE 6:**
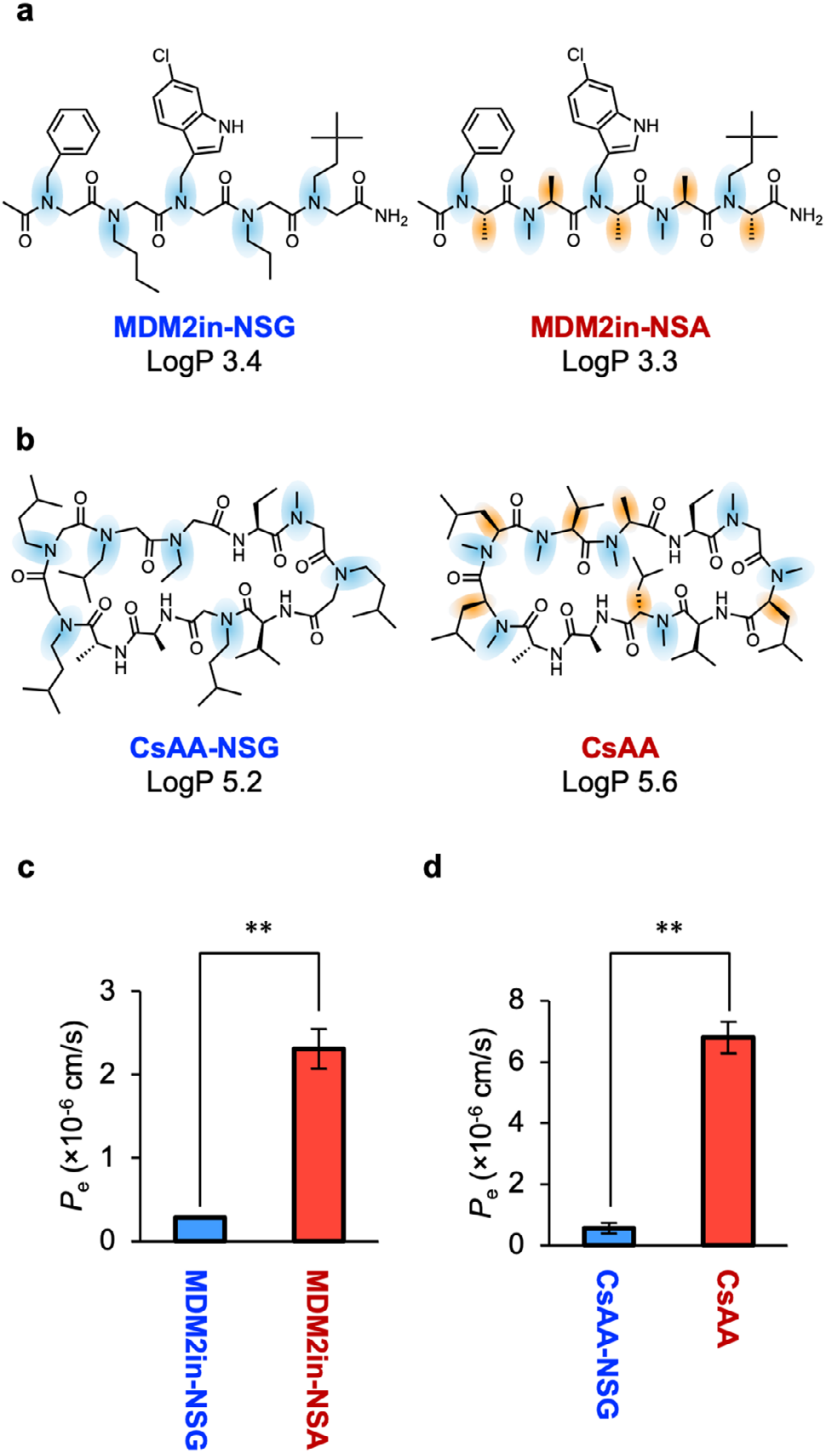
(a) Structures of **MDM2in‐NSG** and **MDM2in‐NSA**. (b) Structures of **CsAA‐NSG** and **CsAA**. (c) *P*_e_ of **MDM2in‐NSG** and **MDM2in‐NSA**. (d) *P*_e_ of **CsAA‐NSG** and **CsAA**. *P*_e_ values represent the mean ± standard deviation of quadruplicate measurements for **MDM2in‐NSG** and **MDM2in‐NSA** and triplicate measurements for **CsAA‐NSG** and **CsAA**. Statistical significance was evaluated using Tukey's test (**p < 0.01).

We first investigated the previously reported MDM2‐inhibiting oligo‐NSA (**MDM2in‐NSA** in Figure 6a) [18]. The *N*‐alkyl peptide strongly binds to MDM2 and exhibits passive membrane permeability. The *N*/*C*α‐dual substitutions in the **MDM2in‐NSA** backbone were previously shown to be important for increasing the binding affinity via conformational rigidification [21]. However, their role in passive membrane permeation has not been investigated. We prepared an oligo‐NSG counterpart of **MDM2in‐NSA** (**MDM2in‐NSG** in Figure 6a) to assess the effect of *N*/*C*α‐dual substitutions on the passive membrane permeability of the bioactive *N*‐alkyl peptide. To compensate for the decrease in lipophilicity of oligo‐NSG caused by the removal of *C*α‐methyl groups, the *N*‐alkyl groups of the second and fourth residues of **MDM2in‐NSG** were extended. PAMPA measurements revealed that **MDM2in‐NSA** exhibited significantly higher membrane permeability than **MDM2in‐NSG** (Figure 6c), which is consistent with the contribution of the backbone steric constraints observed in model *N*‐alkyl peptides.

We also investigated cyclosporin A (CsA) as a model compound. CsA is a membrane‐permeable peptide in which 7 of its 11 backbone amide nitrogens are *N*‐methylated (Figure 1a). Among these *N*‐methyl amino acid residues, six possess *C*α‐substituents; that is, they are non‐glycine residues. Based on the MD analyses described above, suppression of water coordination through conformational restriction and steric hindrance by β‐carbons are expected to facilitate membrane permeation.

To experimentally evaluate this effect, we synthesized CsA derivatives as shown in Figure 6b. To simplify derivative synthesis, we used **CsAA**, an analog in which the Bmt residue of CsA is replaced with alanine, as the parent compound. Because previous studies have shown that this substitution does not reduce passive membrane permeability [30], **CsAA** serves as a suitable framework for evaluating backbone structural effects. We designed a derivative, termed **CsAA‐NSG**, in which the *C*α‐substituents of the *N*‐methyl amino acid residues were removed. To maintain lipophilicity comparable to that of **CsAA**, alkyl groups corresponding to those removed from the α‐carbons were instead introduced as substituents on the amide nitrogens (Figure 6b). PAMPA measurements revealed that **CsAA** exhibited significantly higher membrane permeability than **CsAA‐NSG** (Figure 6d). However, we emphasize that multiple factors, including macrocyclic constraint, intramolecular hydrogen bonding, and side chain structures, contribute to the membrane permeability of CsA. Therefore, the permeability difference between **CsAA** and **CsAA‐NSG** cannot be attributed solely to differences in backbone steric constraints.

These results suggest that even in bioactive *N*‐alkyl peptides, a sterically constrained backbone architecture arising from *N*/*C*α‐dual substitution contributes to high passive membrane permeability.

## 3 Conclusion

In this study, we demonstrate that a sterically constrained backbone architecture is a key determinant of high passive membrane permeability in *N*‐alkyl peptides. Oligo‐NSGs and oligo‐NSAs with similar experimentally determined LogP values exhibited markedly different membrane permeability, indicating that lipophilicity alone does not fully explain the permeability difference between the two peptide formats. This trend is reminiscent of previous cyclic peptide studies showing that the relationship between lipophilicity and permeability can be nonlinear and dependent on peptide scaffold and conformational behavior [24, 25]. Restriction of conformational freedom, together with steric limitation of hydration by β‐carbons, promotes less hydrated conformational states, thereby facilitating membrane permeability (Figure 5).

These findings clarify the critical structural features that enable *N*‐alkyl peptides, an increasingly important class of compounds in drug discovery, to achieve high passive membrane permeability. While *N*‐alkylation of backbone amide bonds has long been recognized to enhance permeability by removing hydrogen‐bond donors and reducing the desolvation energy penalty, the present study reveals that steric constraints generated by the *N*/*C*α‐dually substituted backbone architecture play an essential role.

This insight further suggests that, in natural peptides such as CsA, the prevalence of *N*/*C*α‐dually substituted backbone structures contributes to their high membrane permeability. While a certain degree of conformational flexibility, termed chameleonicity, has been proposed to balance membrane permeability and aqueous solubility in CsA [4, 31, 32, 33], our findings indicate that excessive conformational flexibility can be detrimental to passive membrane permeability. These results suggest that intrinsic backbone steric constraints in *N*‐alkyl peptides are important for promoting low‐hydration states during membrane permeation and thereby enhancing passive membrane permeability.

Moreover, in the context of peptoid‐based drug discovery, where oligo‐NSG lacking *C*α substituents constitutes a fundamental scaffold, the introduction of *C*α‐substituents, as previously explored by Kodadek and coworkers [15] and by our group [16], represents a promising structural design strategy for developing membrane‐permeable peptidomimetics.

Based on these insights, the rational design of membrane‐permeable *N*‐alkyl peptides may facilitate the development of inhibitors targeting intracellular proteins.

## Author Contributions

**Ayumi Inayoshi**: investigation, formal analysis, visualization, writing – original draft, validation. **Mariko Akiba**: conceptualization, methodology, investigation, writing – review and editing. **Marin Yokomine**: methodology, supervision, visualization, writing – original draft. **Masatake Sugita**: methodology, software, investigation, formal analysis, funding acquisition, visualization, writing – original draft, resources. **Koji Umezawa**: methodology, supervision, writing – review and editing. **Yuto Hirota**: methodology, investigation, writing – review and editing. **Yutaka Akiyama**: supervision, resources, writing – review and editing, software, methodology. **Jumpei Morimoto**: project administration, supervision, conceptualization, writing – original draft, funding acquisition, visualization. **Shinsuke Sando**: conceptualization, supervision, project administration, resources, writing – original draft, funding acquisition.

## Conflicts of Interest

The authors declare no conflicts of interest.

## Supporting information



The authors have cited additional references within the Supporting Information [38, 39, 40, 41, 42, 43, 44, 45, 46, 47]. **Supporting File**: anie73930‐sup‐0001‐SuppMat.pdf.

## Data Availability

The data that support the findings of this study are available from the corresponding author upon reasonable request.

## References

[1] J. Chatterjee , C. Gilon , A. Hoffman , and H. Kessler , “N‐Methylation of Peptides: A New Perspective in Medicinal Chemistry,” Accounts of Chemical Research 41 (2008): 1331–1342, 10.1021/ar8000603. 18636716

[2] J. Chatterjee , F. Rechenmacher , and H. Kessler , “N‐Methylation of Peptides and Proteins: An Important Element for Modulating Biological Functions,” Angewandte Chemie International Edition 52 (2013): 254–269, 10.1002/anie.201205674. 23161799

[3] D. S. Nielsen , N. E. Shepherd , W. Xu , A. J. Lucke , M. J. Stoermer , and D. P. Fairlie , “Orally Absorbed Cyclic Peptides,” Chemical Reviews 117 (2017): 8094–8128, 10.1021/acs.chemrev.6b00838. 28541045

[4] K. M. Corbett , L. Ford , D. B. Warren , C. W. Pouton , and D. K. Chalmers , “Cyclosporin Structure and Permeability: From A to Z and Beyond,” Journal of Medicinal Chemistry 64 (2021): 13131–13151, 10.1021/acs.jmedchem.1c00580. 34478303

[5] T. Ono , K. V. Tabata , Y. Goto , et al., “Label‐Free Quantification of Passive Membrane Permeability of Cyclic Peptides Across Lipid Bilayers: Penetration Speed of Cyclosporin A Across Lipid Bilayers,” Chemical Science 14 (2023): 345–349, 10.1039/D2SC05785A. PMC9811578 36687349

[6] P. G. Dougherty , A. Sahni , and D. Pei , “Understanding Cell Penetration of Cyclic Peptides,” Chemical Reviews 119 (2019): 10241–10287, 10.1021/acs.chemrev.9b00008. PMC6739158 31083977

[7] T. R. White , C. M. Renzelman , A. C. Rand , et al., “On‐Resin *N*‐Methylation of Cyclic Peptides for Discovery of Orally Bioavailable Scaffolds,” Nature Chemical Biology 7 (2011): 810–817, 10.1038/nchembio.664. PMC3210067 21946276

[8] C. K. Wang , S. E. Northfield , B. Colless , et al., “Rational Design and Synthesis of an Orally Bioavailable Peptide Guided by NMR Amide Temperature Coefficients,” Proceedings of the National Academy of Sciences of the United States of America 111 (2014): 17504–17509, 10.1073/pnas.1417611111. PMC4267368 25416591

[9] E. Biron , J. Chatterjee , O. Ovadia , et al., “Improving Oral Bioavailability of Peptides by Multiple *N*‐Methylation: Somatostatin Analogues,” Angewandte Chemie International Edition 47 (2008): 2595–2599, 10.1002/anie.200705797. 18297660

[10] M. Weinmüller , F. Rechenmacher , U. K. Marelli , et al., “Overcoming the Lack of Oral Availability of Cyclic Hexapeptides: Design of a Selective and Orally Available Ligand for the Integrin αvβ3,” Angewandte Chemie International Edition 56 (2017): 16405–16409, 10.1002/anie.201709709. 29072809

[11] A. Schumacher‐Klinger , J. Fanous , S. Merzbach , et al., “Enhancing Oral Bioavailability of Cyclic RGD Hexa‐Peptides by the Lipophilic Prodrug Charge Masking Approach: Redirection of Peptide Intestinal Permeability From a Paracellular to Transcellular Pathway,” Molecular Pharmaceutics 15 (2018): 3468–3477, 10.1021/acs.molpharmaceut.8b00466. 29976060

[12] Y. Yamagishi , I. Shoji , S. Miyagawa , et al., “Natural Product‐Like Macrocyclic *N*‐Methyl‐Peptide Inhibitors Against a Ubiquitin Ligase Uncovered From a Ribosome‐Expressed De Novo Library,” Chemistry & Biology 18 (2011): 1562–1570, 10.1016/j.chembiol.2011.09.013. 22195558

[13] M. Tanada , M. Tamiya , A. Matsuo , et al., “Development of Orally Bioavailable Peptides Targeting an Intracellular Protein: From a Hit to a Clinical KRAS Inhibitor,” Journal of the American Chemical Society 145 (2023): 16610–16620, 10.1021/jacs.3c03886. 37463267

[14] A. Ohta , M. Tanada , S. Shinohara , et al., “Validation of a New Methodology to Create Oral Drugs Beyond the Rule of 5 for Intracellular Tough Targets,” Journal of the American Chemical Society 145 (2023): 24035–24051, 10.1021/jacs.3c07145. 37874670

[15] Y. Gao and T. Kodadek , “Synthesis and Screening of Stereochemically Diverse Combinatorial Libraries of Peptide Tertiary Amides,” Chemistry & Biology 20 (2013): 360–369, 10.1016/j.chembiol.2013.01.013. PMC3885906 23521794

[16] J. Morimoto , Y. Fukuda , D. Kuroda , et al., “A Peptoid With Extended Shape in Water,” Journal of the American Chemical Society 141 (2019): 14612–14623, 10.1021/jacs.9b04371. 31405271

[17] R. Kaminker , I. Kaminker , W. R. Gutekunst , et al., “Tuning Conformation and Properties of Peptidomimetic Backbones Through Dual N/Cα‐Substitution,” Chemical Communications 54 (2018): 5237–5240, 10.1039/C8CC01356J. PMC6089238 29726557

[18] Y. Fukuda , M. Yokomine , D. Kuroda , K. Tsumoto , J. Morimoto , and S. Sando , “Peptoid‐Based Reprogrammable Template for Cell‐Permeable Inhibitors of Protein–Protein Interactions,” Chemical Science 12 (2021): 13292–13300. 10.1039/d1sc01560e PMC8528041 34777747

[19] M. Yokomine , J. Morimoto , Y. Fukuda , et al., “Oligo(*N*‐methylalanine) as a Peptide‐Based Molecular Scaffold With a Minimal Structure and High Density of Functionalizable Sites,” Angewandte Chemie International Edition 61 (2022): e202200119, 10.1002/anie.202200119. 35088931

[20] M. A. Dechantsreiter , E. Planker , B. Mathä , et al., “ *N*‐Methylated Cyclic RGD Peptides as Highly Active and Selective αVβ3 Integrin Antagonists,” Journal of Medicinal Chemistry 42 (1999): 3033–3040, 10.1021/jm970832g. 10447947

[21] M. Yokomine , J. Morimoto , Y. Fukuda , et al., “A High‐Resolution Structural Characterization and Physicochemical Study of How a Peptoid Binds to an Oncoprotein MDM2,” Chemical Science 15 (2024): 7051–7060, 10.1039/D4SC01540A. PMC11095393 38756815

[22] R. J. Simon , R. S. Kania , R. N. Zuckermann , et al., “Peptoids: A Modular Approach to Drug Discovery,” Proceedings of the National Academy of Sciences of the United States of America 89 (1992): 9367–9371, 10.1073/pnas.89.20.9367. PMC50132 1409642

[23] In OECD Guideline for the Testing of Chemicals, 2022.

[24] M. R. Naylor , A. M. Ly , M. J. Handford , et al., “Lipophilic Permeability Efficiency Reconciles the Opposing Roles of Lipophilicity in Membrane Permeability and Aqueous Solubility,” Journal of Medicinal Chemistry 61 (2018): 11169–11182, 10.1021/acs.jmedchem.8b01259. 30395703

[25] G. Koch , A. Engstrom , J. Taechalertpaisarn , et al., “Chromatographic Determination of Permeability‐Relevant Lipophilicity Facilitates Rapid Analysis of Macrocyclic Peptide Scaffolds,” Journal of Medicinal Chemistry 67 (2024): 19612–19622, 10.1021/acs.jmedchem.4c01956. PMC11571107 39453819

[26] J. Morimoto , M. Yokomine , Y. Shiratori , et al., “Bottom‐up Design of Peptide Shapes in Water Using Oligomers of *N*‐methyl‐l/d‐alanine,” Chemical Science 16 (2025): 10512–10522, 10.1039/D5SC01483B. PMC12070305 40371367

[27] M. Sugita , S. Sugiyama , T. Fujie , et al., “Large‐Scale Membrane Permeability Prediction of Cyclic Peptides Crossing a Lipid Bilayer Based on Enhanced Sampling Molecular Dynamics Simulations,” Journal of Chemical Information and Modeling 61 (2021): 3681–3695, 10.1021/acs.jcim.1c00380. 34236179

[28] M. Sugita , T. Fujie , K. Yanagisawa , M. Ohue , and Y. Akiyama , “Lipid Composition is Critical for Accurate Membrane Permeability Prediction of Cyclic Peptides by Molecular Dynamics Simulations,” Journal of Chemical Information and Modeling 62 (2022): 4549–4560, 10.1021/acs.jcim.2c00931. PMC9516681 36053061

[29] Y. Hosono , S. Uchida , M. Shinkai , et al., “Amide‐to‐ester Substitution as a Stable Alternative to *N*‐Methylation for Increasing Membrane Permeability in Cyclic Peptides,” Nature Communications 14 (2023): 1416. 10.1038/s41467-023-36978-z PMC10023679 36932083

[30] T. Ono , K. V. Tabata , H. Noji , J. Morimoto , and S. Sando , “Each Side Chain of Cyclosporin A is Not Essential for High Passive Permeability Across Lipid Bilayers,” RSC Advances 13 (2023): 8394–8397, 10.1039/D3RA01358H. PMC10010161 36922944

[31] C. K. Wang , J. E. Swedberg , P. J. Harvey , Q. Kaas , and D. J. Craik , “Conformational Flexibility is a Determinant of Permeability for Cyclosporin,” Journal of Physical Chemistry B 122 (2018): 2261–2276, 10.1021/acs.jpcb.7b12419. 29400464

[32] S. Ono , M. R. Naylor , C. E. Townsend , et al., “Cyclosporin A: Conformational Complexity and Chameleonicity,” Journal of Chemical Information and Modeling 61 (2021): 5601–5613, 10.1021/acs.jcim.1c00771. PMC9531541 34672629

[33] D. Lee , J. Choi , M. J. Yang , C. J. Park , and J. Seo , “Controlling the Chameleonic Behavior and Membrane Permeability of Cyclosporine Derivatives via Backbone and Side Chain Modifications,” Journal of Medicinal Chemistry 66 (2023): 13189–13204, 10.1021/acs.jmedchem.3c01140. 37718494

[34] E. C. Meng , T. D. Goddard , E. F. Pettersen , et al., “UCSF ChimeraX: Tools for Structure Building and Analysis,” Protein Science 32 (2023): e4792. 10.1002/pro.4792 PMC10588335 37774136

[35] T. D. Goddard , C. C. Huang , E. C. Meng , et al., “UCSF ChimeraX: Meeting Modern Challenges in Visualization and Analysis,” Protein Science 27 (2018): 14–25. 10.1002/pro.3235 PMC5734306 28710774

[36] E. F. Pettersen , T. D. Goddard , C. C. Huang , et al., “UCSF ChimeraX: Structure Visualization for Researchers, Educators, and Developers,” Protein Science 30 (2021): 70–82, 10.1002/pro.3943. PMC7737788 32881101

[37] W. Humphrey , A. Dalke , and K. Schulten , “VMD: Visual Molecular Dynamics,” Journal of Molecular Graphics 14 (1996): 33–38, 10.1016/0263-7855(96)00018-5. 8744570

[38] J. D. Hunter , “Matplotlib: A 2D Graphics Environment,” Computing in Science & Engineering 9 (2007): 90–95.

[39] W. H. B. Sauer and M. K. Schwarz , “Molecular Shape Diversity of Combinatorial Libraries: a Prerequisite for Broad Bioactivity,” Journal of Chemical Information and Computer Science 43 (2003): 987–1003, 10.1021/ci025599w. 12767158

[40] Y. Hosono , J. Morimoto , and S. Sando , “A Comprehensive Study on the Effect of Backbone Stereochemistry of a Cyclic Hexapeptide on Membrane Permeability and Microsomal Stability,” Organic & Biomolecular Chemistry 19 (2021): 10326–10331, 10.1039/D1OB02090K. 34821247

[41] Y. Sugita , A. Kitao , and Y. Okamoto , “Multidimensional Replica‐Exchange Method for Free‐Energy Calculations,” Journal of Chemical Physics 113 (2000): 6042–6051, 10.1063/1.1308516.

[42] S. Park , F. Khalili‐Araghi , E. Tajkhorshid , and K. Schulten , “Free Energy Calculation From Steered Molecular Dynamics Simulations Using Jarzynski's Equality,” Journal of Chemical Physics 119 (2003): 3559–3566, 10.1063/1.1590311.

[43] L. Wang , R. A. Friesner , and B. J. Berne , “Replica Exchange with Solute Scaling: A More Efficient Version of Replica Exchange with Solute Tempering (REST2),” Journal of Physical Chemistry B 115 (2011): 9431–9438, 10.1021/jp204407d. PMC3172817 21714551

[44] D. A. Case , H. M. Aktulga , K. Belfon , et al., *Amber 2024*, (University of California,, 2024).

[45] “Molecular Operating Environment (MOE), 2022.02; Chemical Computing Group ULC: Canada” 2022.

[46] W. L. Jorgensen , J. Chandrasekhar , J. D. Madura , R. W. Impey , and M. L. Klein , “Comparison of Simple Potential Functions for Simulating Liquid Water,” Journal of Chemical Physics 79 (1983): 926–935, 10.1063/1.445869.

[47] S. Kumar , J. M. Rosenberg , D. Bouzida , R. H. Swendsen , and P. A. Kollman , “The Weighted Histogram Analysis Method for Free‐Energy Calculations on Biomolecules. I. The Method,” Journal of Computational Chemistry 13 (1992): 1011–1021, 10.1002/jcc.540130812.

