# Supplementary material for: Backbone Steric Constraints Underlie High Passive Membrane Permeability of N‐Alkyl Peptides
Source: Angew Chem Int Ed Engl. 2026 Aug 6;65(41):e3948218. doi: 10.1002/anie.3948218 (PMC13638126; doi:10.1002/anie.3948218)
Supplement: Supplementary file 1 [file ANIE-65-e3948218-s001.pdf]

Supporting Information for

Backbone Steric Constraints Underlie High Passive Membrane Permeability of *N*-Alkyl Peptides

Ayumi Inayoshi, Mariko Akiba, Marin Yokomine, Masatake Sugita, Koji Umezawa, Yuto Hirota, Yutaka Akiyama, Jumpei Morimoto*, Shinsuke Sando*

Table of Contents

Abbreviations 2
Materials and Methods 2
General remarks 2
Synthesis of oligo-NSG 3
Synthesis of oligo-NSAs 3
Synthesis of oligo-NSG libraries 6
Synthesis of oligo-NSA libraries 7
MDM2in-NSA 9
Synthesis of MDM2in-NSG 9
Synthesis of cyclosporin A derivative CsAA 10
Synthesis of cyclosporin A derivative CsAA-NSG 10
Synthesis of heterochiral oligo-NSAs 11
LogP measurement 11
Parallel artificial membrane permeability assay (PAMPA) 12
Caco-2 assay 13
Molecular dynamics (MD) simulations 14
Supplementary Figures 16
FIGURE S1 16
FIGURE S2 17
FIGURE S3 18
FIGURE S4 19
FIGURE S5 20
FIGURE S6 21
FIGURE S7 22
FIGURE S8 23

Abbreviations

ANP, 3-amino-3-(2-nitrophenyl)propionic acid; COMU, ethyl 2-cyano-2-((dimethyliminio)(morpholino)methyloxyimino)acetate hexafluorophosphate; DBU, 1,8-diazabicyclo[5.4.0]undec-7-ene; DCE, 1,2-dichloroethane; DCM, dichloromethane; DDT, 1,1,1-trichloro-2,2-bis(4-chlorophenyl)ethane; DIAD, diisopropyl azodicarboxylate; DIC, *N*,*N'*-diisopropylcarbodiimide; DIPEA, *N*,*N*-diisopropylethylamine; DMF, *N*,*N*-dimethylformamide; DMSO, dimethyl sulfoxide; HFIP, 1,1,1,3,3,3-hexafluoro-2-propanol; HOAt, 1-hydroxy-7-azabenzotriazole; NMP, *N*-methyl-2-pyrrolidone; *o*-Ns-Cl, 2-nitrobenzenesulfonyl chloride; Oxyma, ethyl cyano(hydroxyimino)acetate; PyAOP, (7-azabenzotriazol-1-yloxy)tripyrrolidinophosphonium hexafluorophosphate; TFA, trifluoroacetic acid; TIPS, triisopropylsilane; TMP, 2,4,6-trimethylpyridine.

Materials and Methods

General remarks

Chemicals and solvents used in this study were purchased from commercial suppliers and used without further purification. Preparative high performance liquid chromatography (HPLC) was performed on a Prominence HPLC system (Shimadzu) with a 5C18-AR-II column (Nacalai tesque, 10 mm I.D.×150 mm, 34350-41). Liquid chromatography–mass spectrometry (LC-MS) and ultra-performance liquid chromatography (UPLC) were performed on an ACQUITY UPLC H-Class/SQD2 (Waters) using an ACQUITY UPLC BEH C_{18} Column (Waters, 1.7 µm, 2.1 mm × 50 mm) and an ACQUITY^{TM} Premier system (Waters Corp., Milford, MA, USA) coupled to a Waters Xevo^{TM} G3 QTof mass spectrometer using an ACQUITY Premier Peptide BEH C18 Column (130 Å, 1.7 µm, 2.1 × 100 mm). High-resolution mass spectrometry (HRMS) data were acquired using a micrOTOF II instrument (Bruker Daltonics). All quantum chemical calculations were carried out with the Gaussian16 package. Distilled water was further purified with Millipore Milli-Q (Milli-Q Reference, Merck). Some plots were generated using the Python library Matplotlib.[38]

Principal moment of inertia (PMI) analysis

The PMI analysis was conducted according to a previously reported method.[39] All atoms, including hydrogen atoms, were included in the analysis. Model structures of NMA pentamers were generated as follows. First, the most stable conformer of L-NMA residues (φ = –135°, ψ = 75°) and D-NMA residues (φ = 135°, ψ = –75°) on the Ramachandran-type plot were connected to construct each pentamer, and the termini were capped with an acetyl group at the N-terminus and an amide group at the C-terminus. Substituents on the amide nitrogens were introduced with χ angles considered favorable based on previous DFT calculations and molecular dynamics simulations of oligo-NSAs.[16,19,26] The generated conformers were energy minimized by molecular mechanics using the MMFF94s force field.

Synthesis of oligo-NSG

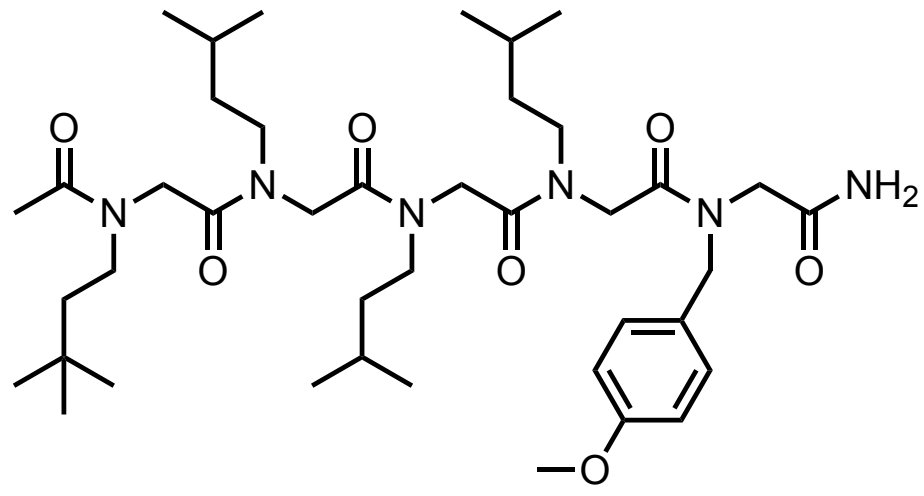

NSG1

Resin preparation

Rink Amide MBHA resin (0.41 mmol/g, 12 μmol per peptide) was swollen in DMF in a fritted syringe. After removal of DMF, the resin was treated twice with 20% piperidine/DMF (3 min and 12 min), followed by washing with DMF (×3).

Elongation of peptide chain

A solution of bromoacetic acid (BAA) (20 equiv., 1 M) and DIC (10 equiv., 1 M) in DMF was preincubated for 5 min and then added to the resin. The resin was incubated at 37 °C for 1 h with continuous shaking and washed with DMF (×3). A solution of a primary amine (20 equiv., 1 M) in DMF was then added, and the resin was incubated at 37 °C for 1–2 h with continuous shaking, followed by washing with DMF (×3).

Acetyl capping

The oligomer was acetyl-capped by incubating the resin overnight with a solution of acetic anhydride (10 equiv., 0.5 M) and DIPEA (20 equiv., 1.0 M) in DMF. The resin was washed with DMF and DCM (×3 each).

Peptide cleavage from resin and purification

The resin was swollen in DCM and treated with TFA/TIPS/water (95:2.5:2.5, 1 mL) for 1 h. The resin was washed (×2 each) with TFA and MeOH. After solvent removal, the crude product was purified by HPLC.

Yield 55%. HRMS (ESI-TOF MS) *m/z*: $[M+H]^+$ calcd for $C_{41}H_{71}N_6O_7^+$ 759.54; found 759.86.

Synthesis of oligo-NSAs

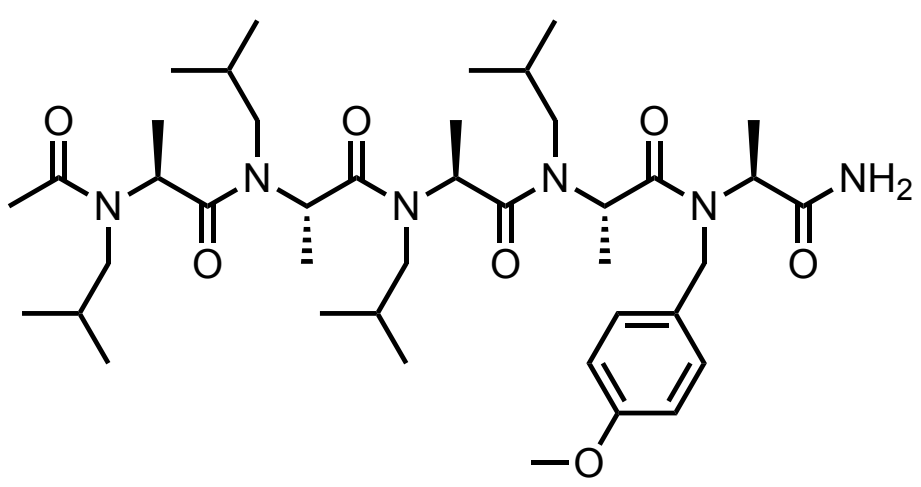

NSA2

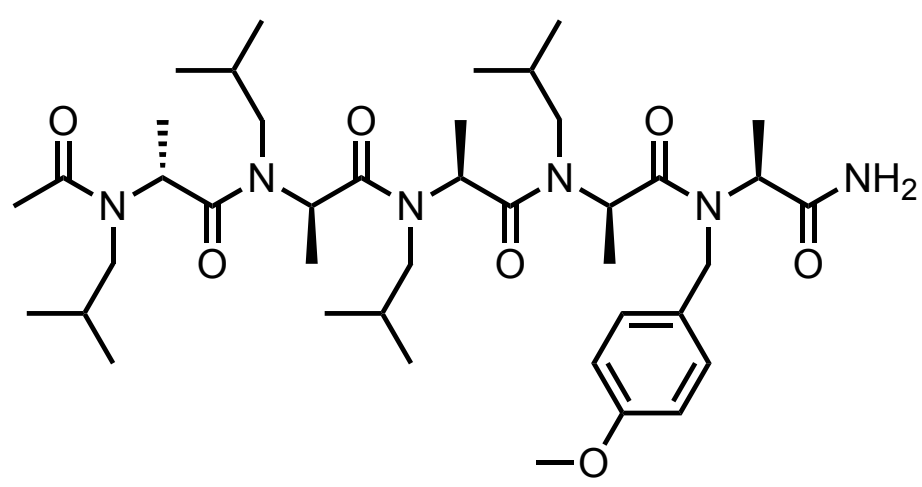

NSA3

Resin preparation

Fmoc-NH-Sieber resin (0.46 mmol/g, 10 μmol per peptide) was swollen in DMF in a fritted syringe. After removal of DMF, the resin was treated twice with 20% piperidine/DMF (3 min and 12 min) and washed with DMF (×3).

Coupling reaction of the 1st residue

Fmoc-L-alanine (4 equiv., 0.2 M), COMU (4 equiv., 0.2 M), and DIPEA (8 equiv., 0.4 M) were dissolved in DMF and added to the resin. The resin was incubated for 40 min with continuous shaking and then washed with DMF (×3).

Introduction of an *N*-alkyl group at the 1st residue (reductive amination)

After removal of Fmoc, the resin was incubated with *p*-anisaldehyde (20 equiv., 1 M) in DMF for 3 h, followed by washing with DMF and DCM (×3 each). $NaBH_4$ (10 equiv.) and DCM/MeOH (3:1, 1 mL per 50 μmol resin loading) were added, and the resin was incubated for 30 min with the syringe uncapped. The resin was washed with MeOH, DCM, and DMF (×3 each).

Coupling reaction of the 2nd residue

Fmoc-L-alanine (4 equiv., 0.2 M), DIC (8 equiv., 0.4 M), and Oxyma (4 equiv., 0.2 M) were dissolved in DMF/DCE (1:1) and preactivated for 30 min. DIPEA (0.4 equiv., 0.02 M) was added, and the solution was applied to the resin. The resin was incubated for 3 h at 60 °C with continuous shaking and washed with DMF (×3). The coupling reaction was repeated one additional time.

Introduction of an *N*-alkyl group at the 2nd residue (reductive amination)

After Fmoc removal, the resin was incubated with isobutyraldehyde (20 equiv., 1 M) in DMF for 1 h. After washing with DMF and DCM (×3 each), $NaBH_4$ (10 equiv.) and DCM/MeOH (3:1, 1 mL per 50 μmol resin loading) were added and the resin was incubated for 30 min with the syringe uncapped. The resin was washed with MeOH, DCM, and DMF (×3 each).

Elongation of 3rd–5th residue

Coupling and deprotection cycles were repeated to synthesize the oligomer on the resin.

Acetyl capping

The oligomer was acetyl-capped by incubating the resin with acetic anhydride (10 equiv., 0.5 M) and DIPEA (20 equiv., 1.0 M) in DMF for 24 h. The resin was washed with DMF and DCM (×3 each).

Peptide cleavage from resin and purification

The resin was treated with 1% TFA/DCM (500 μL) for 1 min, repeated four additional times. The cleavage solution was immediately transferred into a vial containing 10% pyridine/MeOH (1.25 mL).

The resin was washed with MeOH (×3), and the washings were combined. After solvent removal, the crude product was purified by HPLC.

Oligo-NSA **2**: Yield 7%. MS (ESI-TOF MS) *m/z*: $[M+Na]^+$ calcd for $C_{41}H_{70}N_6O_7Na^+$ 781.52; found 781.89.
Oligo-NSA **3**: Yield 6%. MS (ESI-TOF MS) *m/z*: $[M+Na]^+$ calcd for $C_{41}H_{70}N_6O_7Na^+$ 781.52; found 782.05.

Synthesis of oligo-NSG libraries

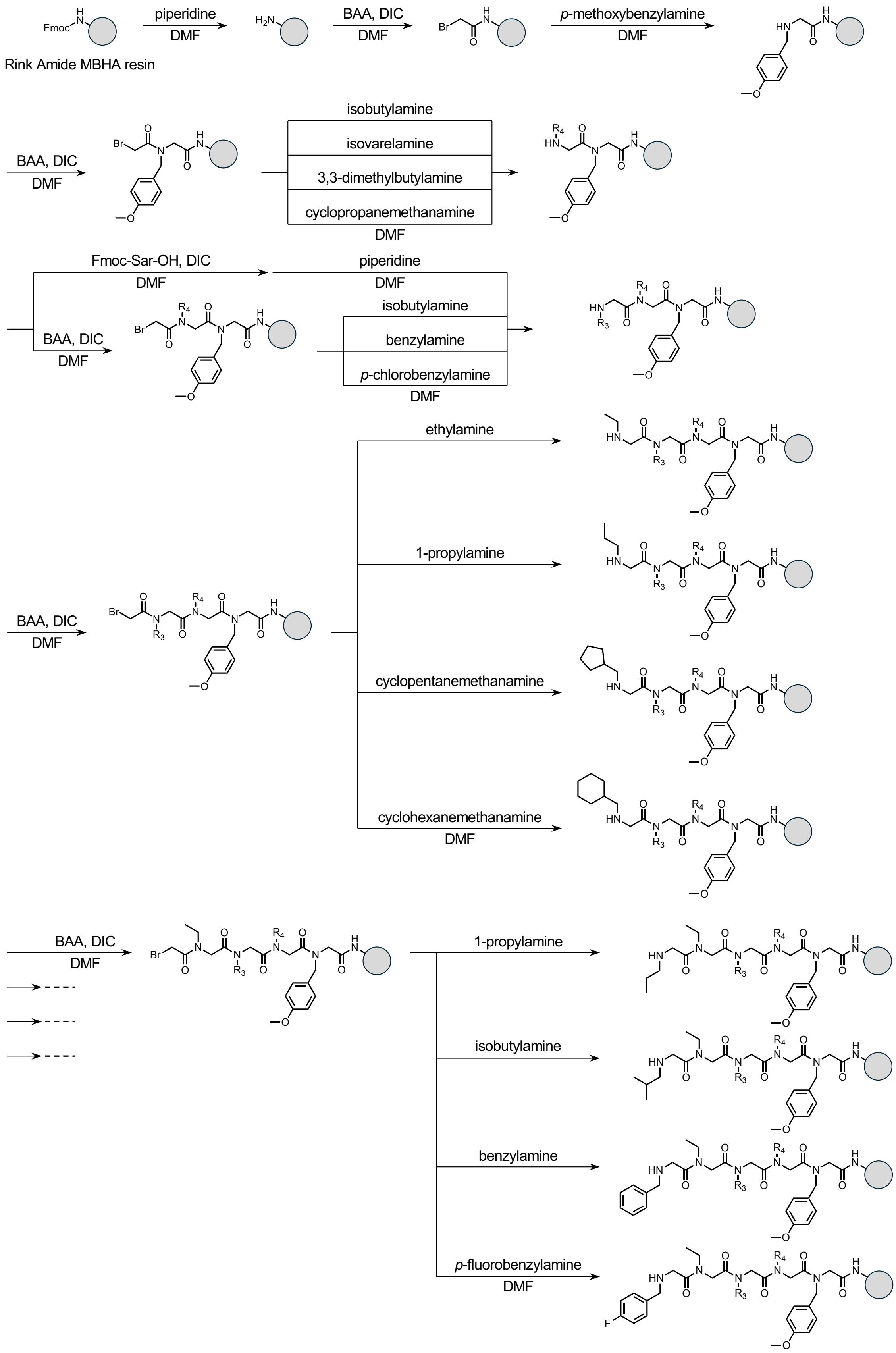

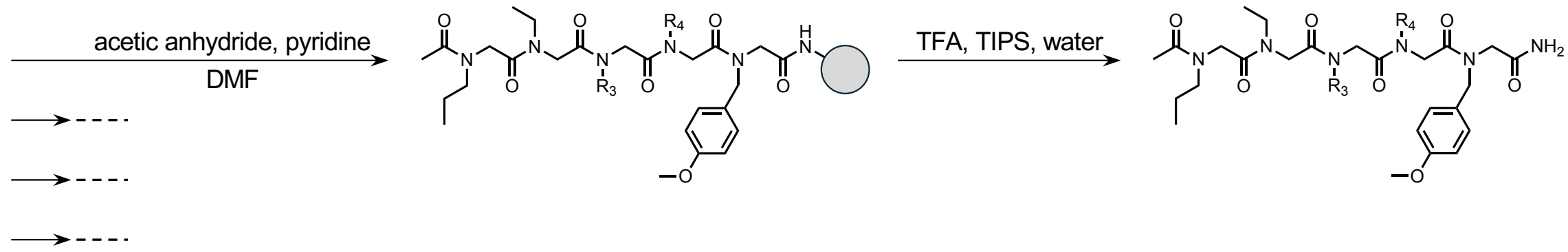

Oligo-NSG libraries were synthesized according to the procedures described in the "**Synthesis of oligo-NSG**" section with the following modifications. Rink Amide MBHA resin (0.76 mmol/g) was used at a loading of 0.79 µmol per peptide, corresponding to a total of 201.55 µmol for 256 sequences. Reactions with bromoacetic acid and subsequent amine displacement were conducted at room temperature. Acetyl capping was conducted using acetic anhydride (20 equiv., 0.5 M) and pyridine (40 equiv., 1.0 M) in DMF for 3 h. After cleavage and lyophilization, the peptides were dissolved in DMSO, and the overall concentrations were determined based on the UV absorbance at 280 nm. For incorporation of the Sar residue, coupling of Fmoc-Sar-OH was performed instead of bromoacetic acid coupling followed by amine displacement. Specifically, Fmoc-Sar-OH (10 equiv., 1.0 M) and DIC (5 equiv., 0.5 M) were dissolved in DMF and preactivated for 5 min before addition to the resin. The resin was incubated for 2 h with continuous shaking. After washing with DMF (×3), Fmoc deprotection was conducted.

Synthesis of oligo-NSA libraries

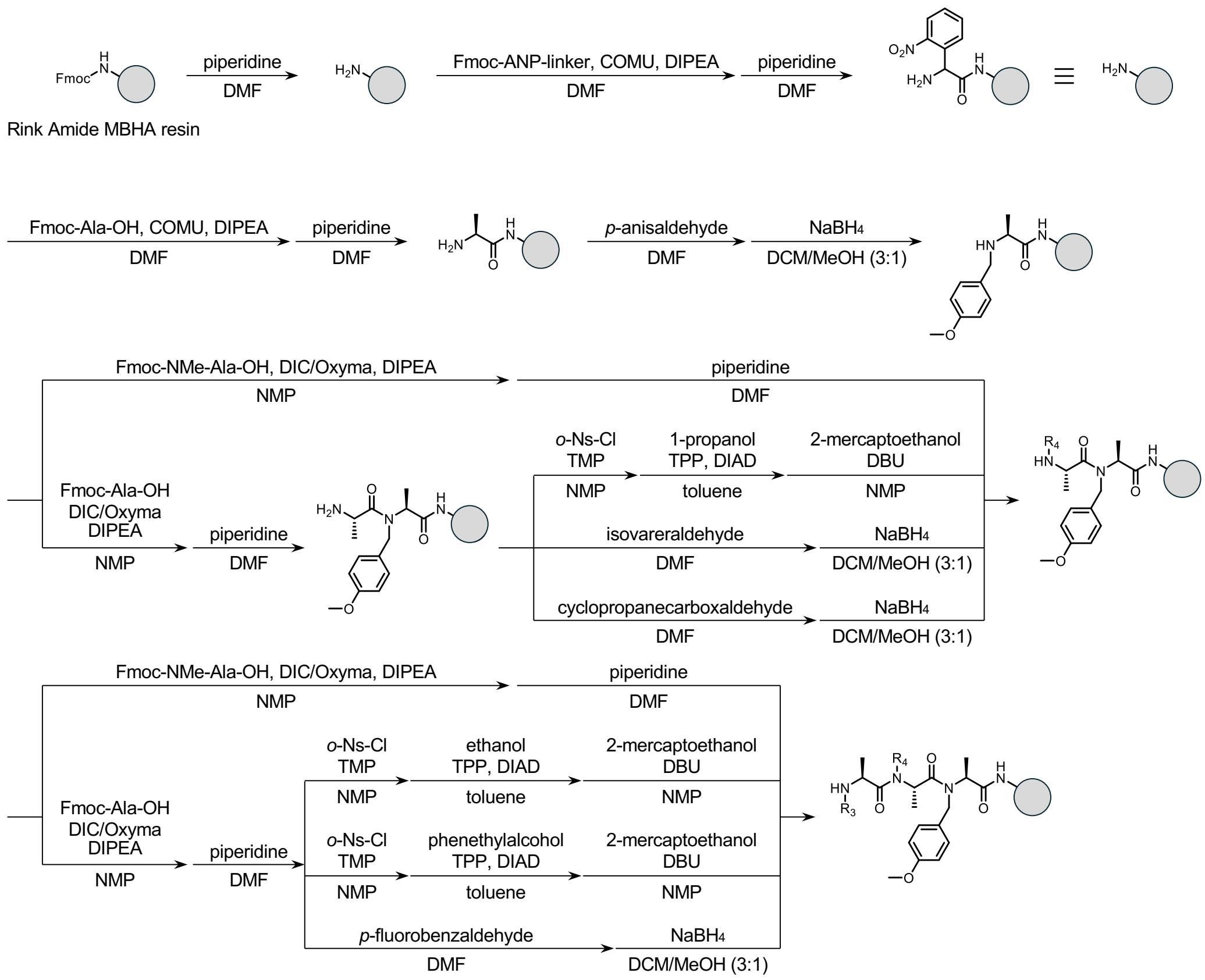

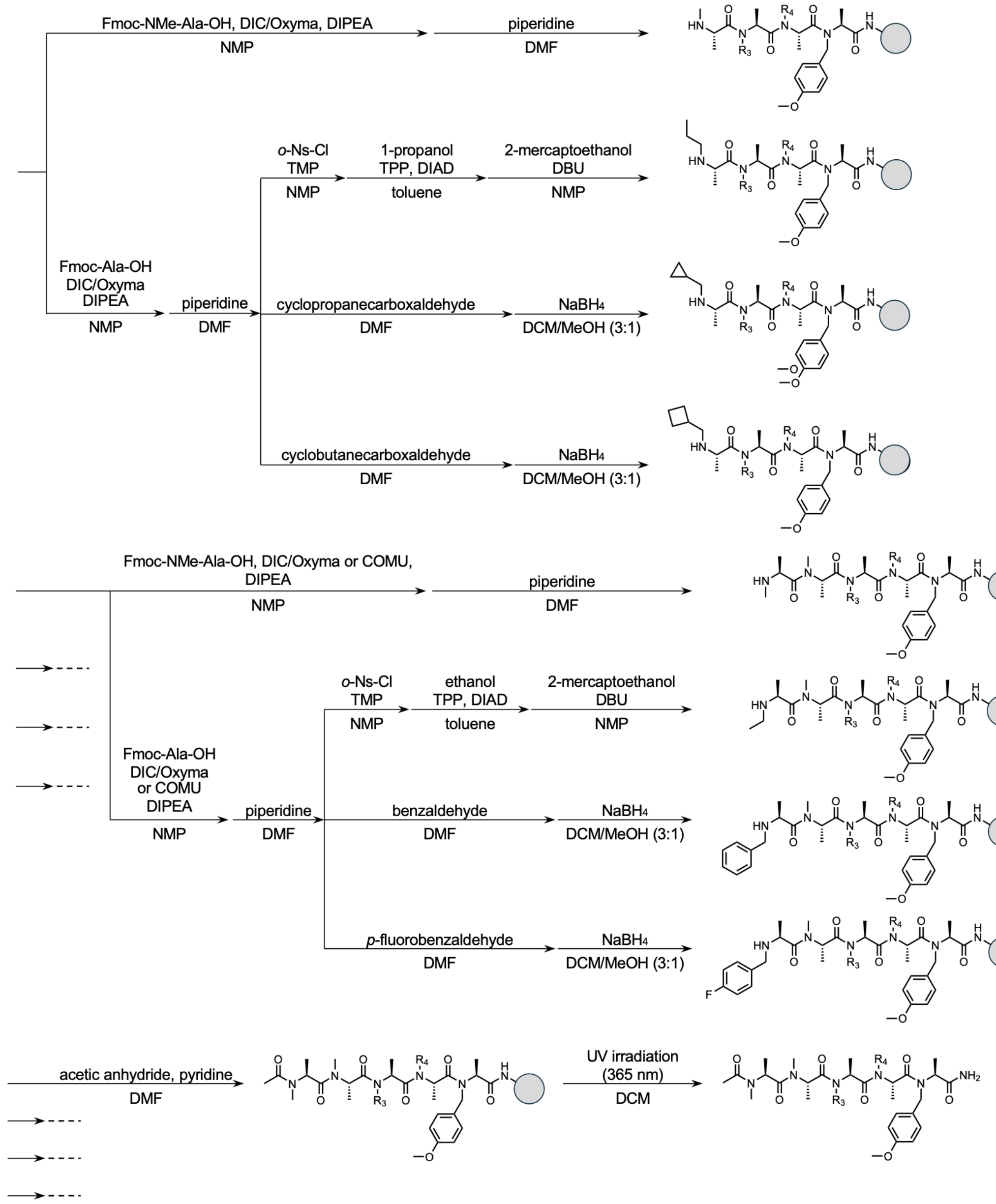

Oligo-NSA libraries were synthesized according to the procedures described in the "**Synthesis of oligo-NSAs**" section with the following modifications. Rink Amide MBHA resin (0.76 mmol/g) was used at a loading of 0.82 μmol per peptide, corresponding to a total of 209.00 μmol for 256 sequences. Oligo-NSAs bearing a cyclopropylmethyl group at R_4 were not detected by LC-MS during PAMPA experiments, suggesting unsuccessful residue incorporation. Therefore, the cyclopropylmethyl substituent at R_4 was excluded from Figure 2d. The total number of sequences was 192 after excluding unsuccessful synthesis. The coupling reaction of the 1st residue was conducted for 1.5 h. *N*-Methyl groups were introduced by coupling Fmoc-*N*-methyl-L-alanine using DIC and Oxyma. *N*-Ethyl, *N*-propyl, and *N*-phenethyl groups were introduced via the Fukuyama–Mitsunobu reaction described below, whereas other substituents were introduced by reductive amination as described in the "**Synthesis of oligo-NSAs**" section. In reductive amination, aldehyde treatment and $NaBH_4$ reduction were conducted for 1.5 h and 1 h, respectively. Coupling reactions using DIC and Oxyma were conducted in NMP. Coupling steps following *N*-methyl-L-alanine incorporation were conducted

using COMU and DIPEA instead of DIC and Oxyma. Acetyl capping was performed using acetic anhydride (20 equiv., 0.5 M) and pyridine (40 equiv., 1.0 M) in DMF for 3 h at 60 °C.

Coupling reaction of the ANP linker

Fmoc-ANP-linker (4 equiv., 0.2 M), COMU (4 equiv., 0.2 M), and DIPEA (8 equiv., 0.4 M) were dissolved in DMF and applied to the resin. The resin was incubated for 1.5 h with continuous shaking and washed with DMF (×3).

Introduction of *N*-ethyl, *N*-propyl and *N*-phenethyl groups (Fukuyama–Mitsunobu reaction)

After Fmoc removal, the resin was washed with NMP (×3) and treated with *o*-Ns-Cl (4 equiv., 0.1 M) and TMP (10 equiv., 0.25 M) in NMP for 15 min. The resin was washed with NMP and toluene (×3 each). A solution of alcohol (20 equiv., 0.8 M) and triphenylphosphine (10 equiv., 0.4 M) in toluene was added, followed by dropwise addition of DIAD (10 equiv., 0.4 M) in toluene to afford final concentration of 0.4 M alcohol, 0.2 M triphenylphosphine and 0.2 M DIAD. The reaction vessel was capped and shaken for 3 h at 60 °C. After washing with toluene and NMP (×3 each), the *o*-Ns group was removed by treatment with 2-mercaptoethanol (10 equiv., 0.4 M) and DBU (5 equiv., 0.2 M) in NMP for 8 min. This deprotection step was repeated two additional times, followed by washing with NMP and DMF (×3 each).

Peptide cleavage from resin by UV irradiation

The resin was swollen in DCM in a vial and irradiated with UV light (365 nm) for 2 h with manual agitation every 20 min. The resin was washed with DCM and MeOH (×3 each). The cleaved solution and washings were combined and lyophilized. The peptides were dissolved in DMSO, and overall concentrations were determined from the UV absorbance at 280 nm.

MDM2in-NSA

The peptide synthesized in our previous report[18] was used.

MDM2in-NSA: MS (ESI-TOF MS) *m/z*: $[M+Na]^+$ calcd for $C_{41}H_{58}ClN_7O_6Na^+$ 802.4; found 802.8.

Synthesis of MDM2in-NSG

The peptide was synthesized on resin according to the procedure described in the section "**Synthesis of oligo-NSG**". The *N*-neohexyl and *N*-(6-chloroindolylmethyl) groups were introduced by reductive amination according to the procedure previously described for the synthesis of **MDM2in-NSA**.[18]

Peptide cleavage from the resin and purification

The resin was treated with 1% TFA/DCM (1 mL) for 2 min, and this treatment was repeated four additional times. Each cleavage solution was immediately transferred to a vial containing 10% pyridine/MeOH (3 mL). After removal of the solvents, the crude product was purified by HPLC using 20 mM ammonium acetate (pH 7.0) and acetonitrile as solvents.

Yield

After HPLC purification, 0.17 µmol of the peptide was obtained (0.7% yield).

MDM2in-NSG: MS (ESI-TOF MS) *m/z*: $[M+H]^+$ calcd for $C_{41}H_{59}ClN_7O_6^+$ 780.4; found 781.0.

Synthesis of cyclosporin A derivative CsAA

CsAA was synthesized as previously reported[30] with minor modifications. Amino acid coupling reactions were performed using Fmoc-aa-OH (3 equiv., 0.2 M), COMU (3 equiv., 0.2 M), and DIPEA (6 equiv., 0.4 M) in DMF. Cyclization was conducted overnight at room temperature.

Yield

After HPLC purification, 49 µmol of peptide was obtained (36% yield).

CsAA: MS (ESI-TOF MS) *m/z*: $[M+H]^+$ calcd for $C_{56}H_{102}N_{11}O_{11}^+$ 1104.8; found 1105.0.

Synthesis of cyclosporin A derivative CsAA-NSG

Fmoc-L-Ala-OH was loaded onto polystyrene resin bearing a chlorotrityl chloride (CTC) linker by incubating the resin with a DCM solution of Fmoc-L-Ala-OH (2 equiv., 0.1 M) and DIPEA (4 equiv., 0.2 M) for 2 h at room temperature. After washing with DCM (×3), the resin was treated with DCM/MeOH/DIPEA (17:2:1) for 15 min. Following additional washing with DCM (×3), the remaining peptide sequence was synthesized using the procedures described below.

Introduction of an NSG residue

Bromoacetic acid (2 M in DMF, 250 µL, 20 equiv.) and DIC (1 M in DMF, 250 µL, 10 equiv.) were mixed and preincubated for 5 min before addition to the resin. The reaction vessel was shaken for 1 h at room temperature, and the resin was washed with DMF (×3). A primary amine (1 M in DMF, 500 µL, 20 equiv.) was added, and the resin was incubated for 1 h at room temperature with continuous shaking, followed by washing with DMF (×3).

Introduction of an amino acid residue

Fmoc-aa-OH (4 equiv., 0.2 M), DIC (5.6 equiv., 0.28 M), and Oxyma (2.5 equiv., 0.125 M) in NMP (500 µL) were added to the resin, and the reaction vessel was incubated for 1–3 h at room temperature or at 60 °C.

Cleavage from resin

After the final Fmoc deprotection, the resin was washed with DCM (×3) and treated with 30% HFIP/DCM (1 mL) for 20 min at room temperature. The treatment was repeated three additional times. The combined cleavage solution and MeOH washings were evaporated.

Cyclization

The residue was dissolved in DMSO (200 µL), followed by addition of DCM (500 µL). A solution containing PyAOP (2 equiv.), HOAt (2 equiv.), and DIPEA (10 equiv.) in DCM (700 µL) was added, and the mixture was incubated overnight at room temperature. After solvent removal, the residue was dissolved in 60% aqueous MeCN (2 mL) and purified by HPLC.

Yield

After HPLC purification, 5.16 µmol of peptide was obtained (21% yield).

CsAA-NSG: MS (ESI-TOF MS) *m/z*: $[M+H]^+$ calcd for $C_{56}H_{102}N_{11}O_{11}^+$ 1104.8; found 1105.2.

Synthesis of heterochiral oligo-NSAs

Heterochiral oligo-NSAs were synthesized according to the procedures described in the "**Synthesis of oligo-NSAs**" section. The observed mass values are summarized below.

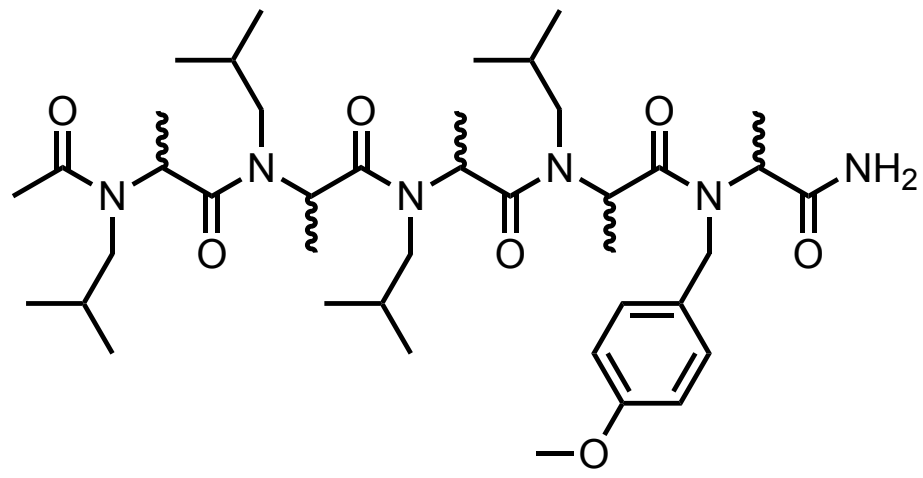

	[M+Na]$^+$	
	Calcd.	Obsd.
DLLLL	781.52	781.89
LDLLL	781.52	781.94
LLDLL	781.52	781.94
LLLDL	781.52	781.89
DDLLL	781.52	782.10
DLDLL	781.52	781.89
DLLDL	781.52	781.89
LDDLL	781.52	781.89
LDLDL	781.52	781.94
LLDDL	781.52	781.94
DDDLL	781.52	782.10
DDLDL	781.52	782.05
DLDDL	781.52	781.89
LDDDL	781.52	781.94
DDDDL	781.52	782.05

LogP measurement

Log P values were determined by LC-MS using an octadecyl column. Calibration and determination were performed according to OECD guideline No. 117.[23] Thiourea was used as a t_0 standard. Standard compounds used for calibration are shown in the corresponding calibration curves.

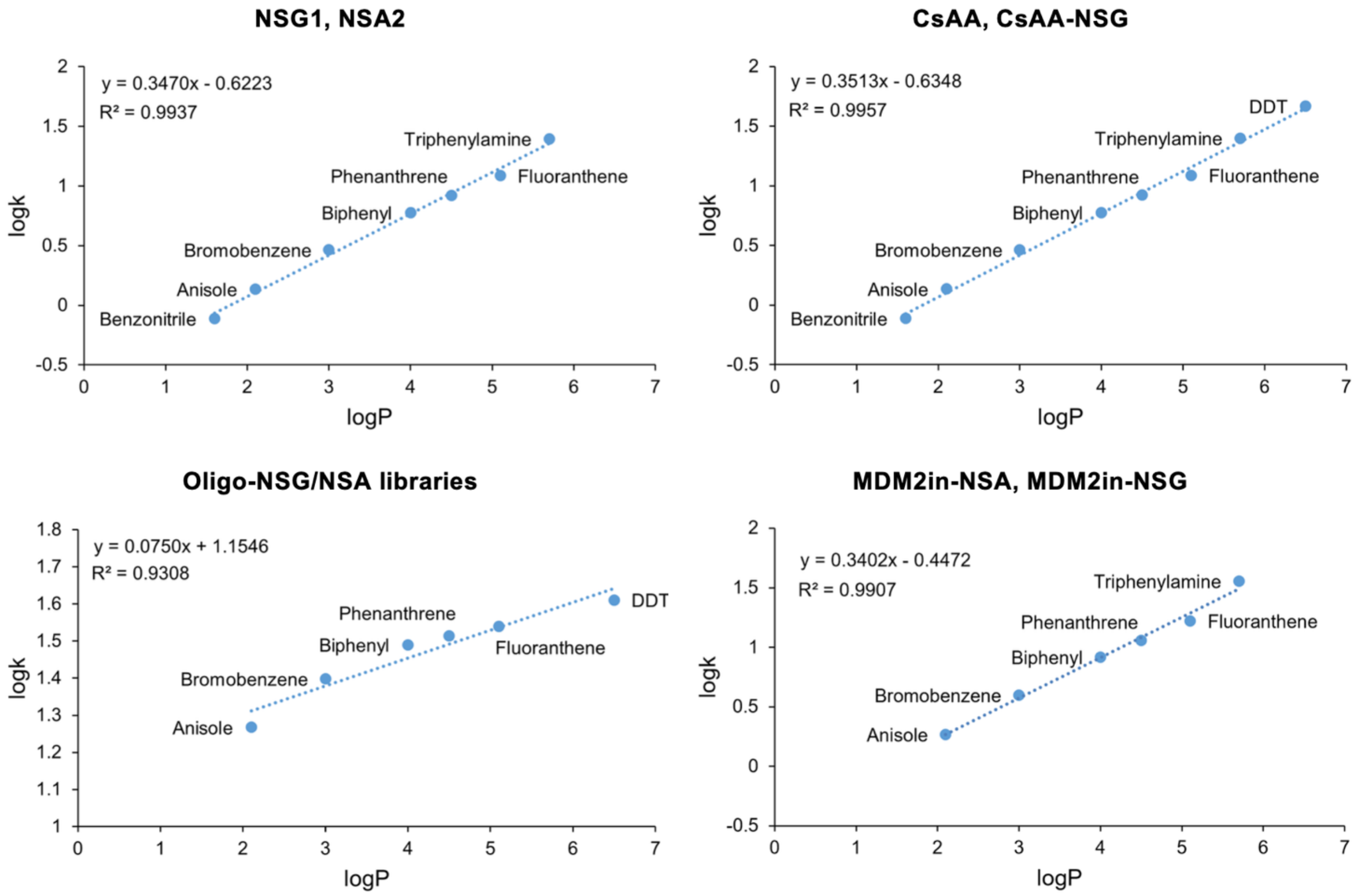

Parallel artificial membrane permeability assay (PAMPA)

PAMPA experiments were conducted as previously described[40] with minor modifications. For single-compound per well analysis, each compound was tested at 10 µM, except for **MDM2in-NSA** and **MDM2in-NSG**, which were tested at 5 µM. For library analysis, each sub-library was tested at an overall concentration of 100 µM. Incubation was conducted for 4 h at 37 °C. Calibration curves are shown below. For **CsAA-NSG**, two samples fell outside the calibration range and were therefore extrapolated (orange points in the graph). For library analysis, data were excluded if (i) the donor or acceptor responses were on the same order of magnitude as the blank or $\leq 10^3$, or (ii) the R value was below –0.5. As a result, the data of 230 oligo-NSGs and 150 oligo-NSAs were included in the analysis.

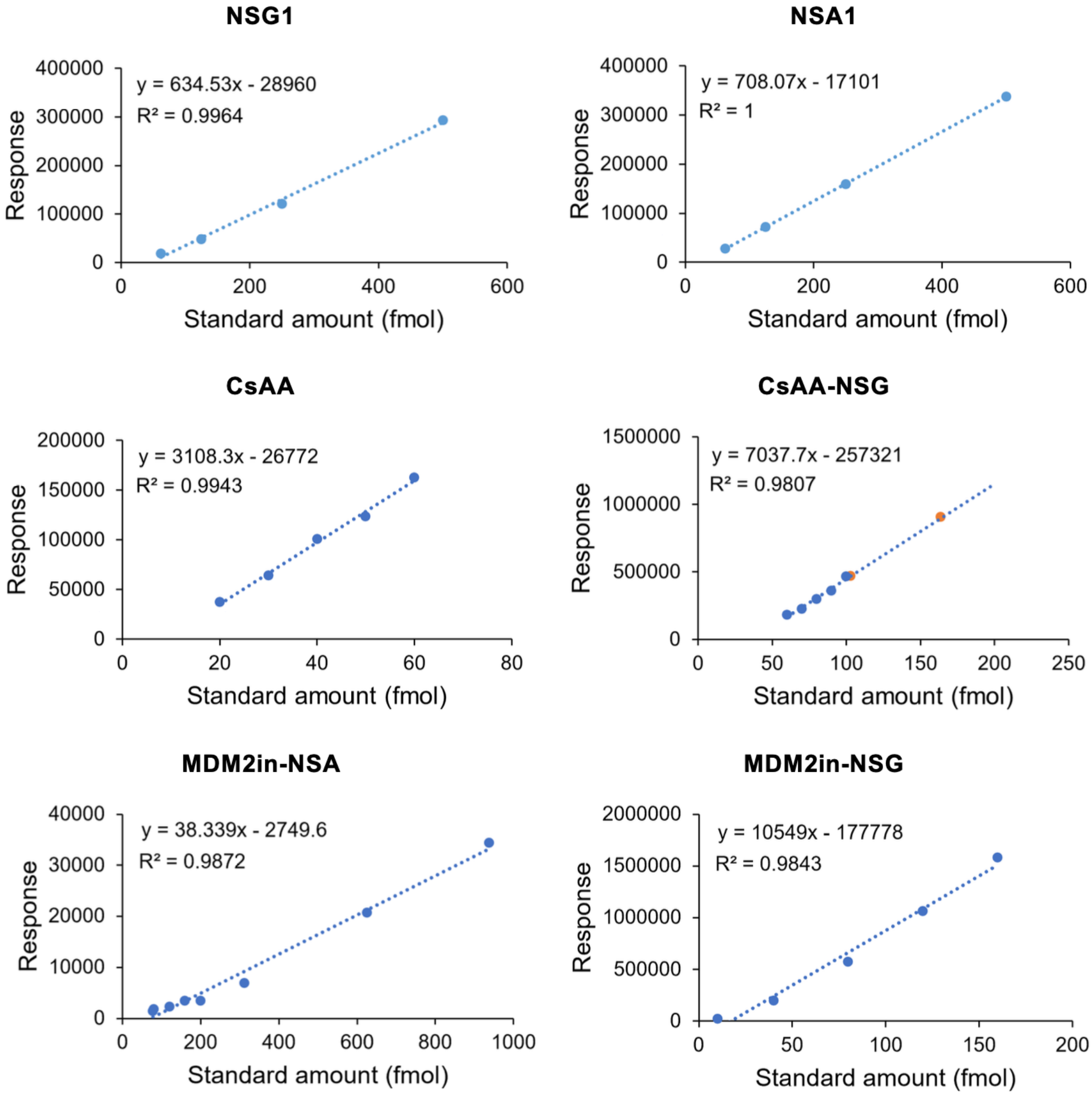

Caco-2 assay

Caco-2 assay was performed as previously described[29] with minor modifications. For single-compound per well analysis, compounds were tested at 10 µM. Incubation was conducted for 2 h at 37 °C. Verapamil (100 µM) was used as a transporter inhibitor. **NSG1** was assayed concurrently with **NSA2** and **NSA3** in separate experiments and was therefore measured twice. The value reported for **NSG1** is the mean of the two independent assays.

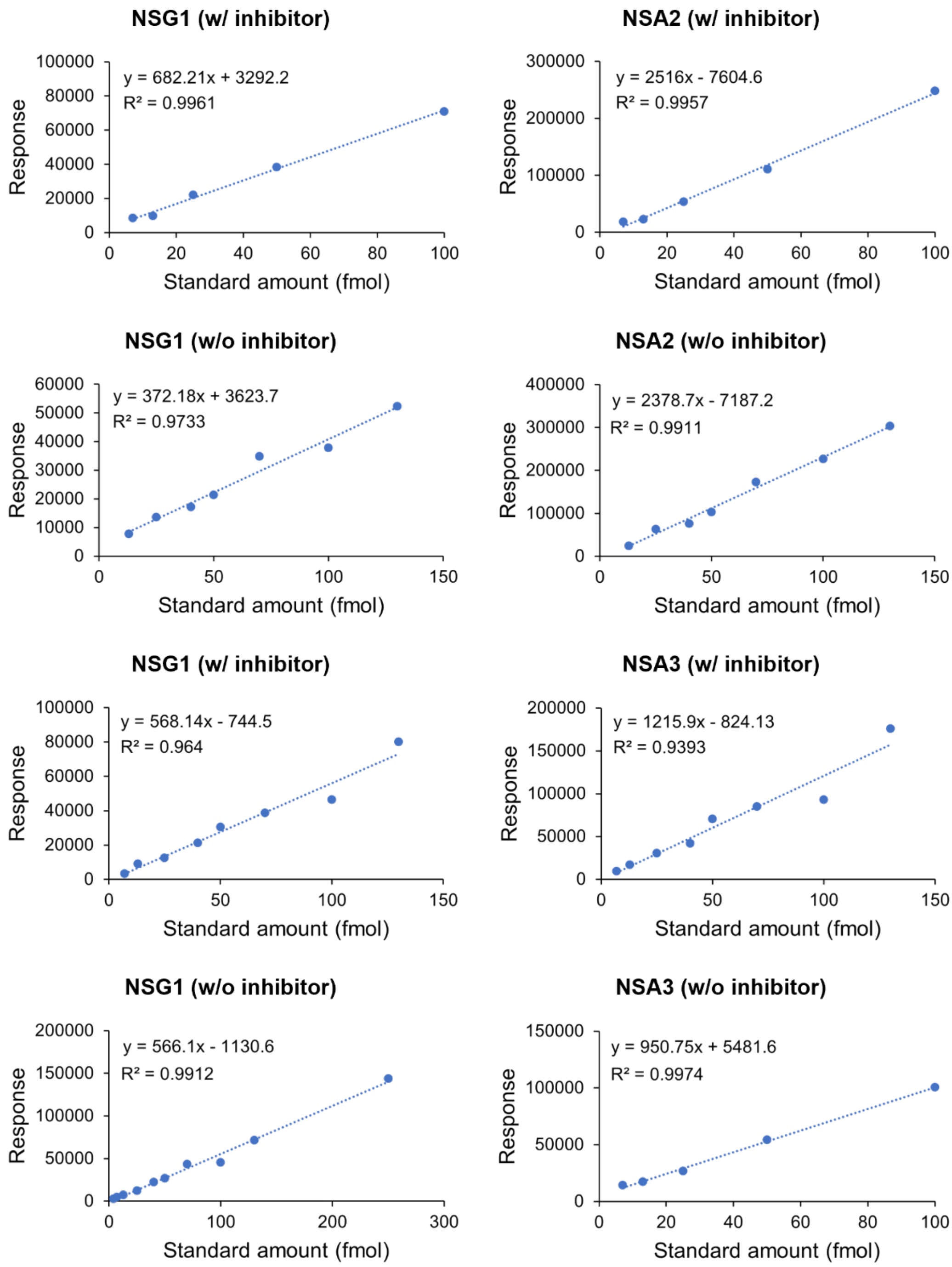

Molecular dynamics (MD) simulations

MD simulations for exploring the membrane permeation process were performed as previously described[28] with minor modifications. We used a membrane model composed of 1-palmitoyl-2-oleoyl-sn-glycero-3-phosphocholine (POPC) with 40 mol% cholesterol. Sugita et al. showed that this model provides optimal performance for predicting membrane permeability when combined with the force-field parameter set described below.[28] The REST/REUS simulation[41] was carried out with 224 replicas consisting of 28 windows with different harmonic restraint centers and 8 windows with different solute temperature. The reaction coordinate z is defined as the distance on the axis orthogonal to the membrane plane between the center of mass of all nitrogen atoms of

phosphatidylcholine and that of backbone amide nitrogen atoms. The peptide molecule is defined as the solute region. The restraint center and force constant of the harmonic potentials were set to 1.0 Å intervals and 1.5 kcal/mol/Å^2 for the section from z = 0 Å to 6 Å, to 1.5 Å intervals and 0.5 kcal/mol/Å^2 for the section from z = 6.0 to 30.0 Å, and to 3.0 Å intervals and 0.2 kcal/mol/Å^2 for the section from z = 33.0 to 45.0 Å. The solute temperature for each replica was set to 300 K, 340 K, 390 K, 455 K, 540 K, 645 K, 785 K, and 980 K. A 300-ns production run was performed after 200 ns of equilibration. Exchanges between adjacent replicas were attempted every 10 ps based on the Metropolis scheme. The initial coordinates were extracted from the trajectory of pre-executed steered MD[42] with solute tempering.[43] The center of mass of the backbone amide nitrogen atoms was pulled from z = 45.0 Å to –5.0 Å on the reaction coordinate. A pulling rate of 0.36 Å/ns and force constant of 3.0 kcal/mol/Å^2 were used. In this process, the solute temperature is set to 2,100 K to obtain diverse conformations. All MD simulations were performed using the GPU accelerated PMEMD module (pmemd.cuda) of the AMBER 24 software package.[44] Peptide and POPC molecules were parameterized with Amber10:EHT parameter set in the MOE[45] and Lipid 17[44] force field. The TIP3P water model[46] was used. Free energy profiles along the reaction coordinate were estimated based on the weighted histogram analysis method.[47] Peptide conformations were analyzed by principal component analysis based on the main-chain dihedral angles (phi, psi, omega). Thirty-dimensional eigenvectors were obtained by diagonalizing the variance-covariance matrix calculated from all snapshots of **NSG1** and **NSA2** with the temperature of 300 K. Subsequently, trajectories corresponding to the inside and outside of the lipid membrane of each peptide were projected onto the first and second principal components (total contribution is 37%). The representative structures in solution phase of the **NSG1** and **NSA2** were determined as the centroid of the largest cluster after clustering the conformations into 10 clusters using the k-means method.

Supplementary Figures

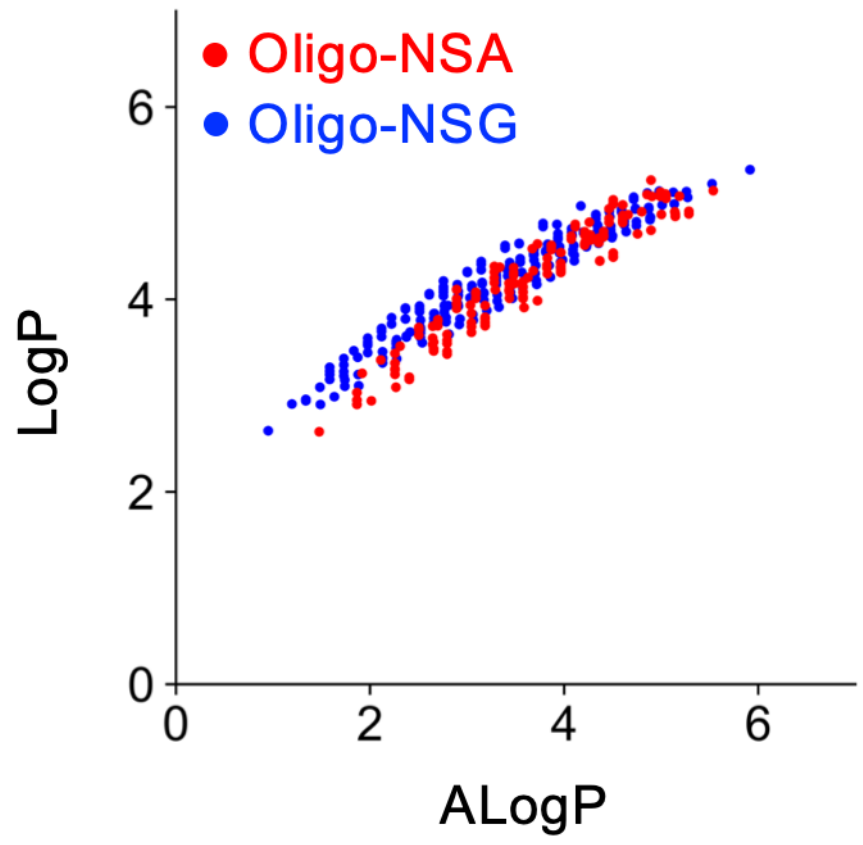

FIGURE S1 | The correlation between ALogP and chromatographically determined LogP for the oligo-NSG (blue) and oligo-NSA (red) libraries.

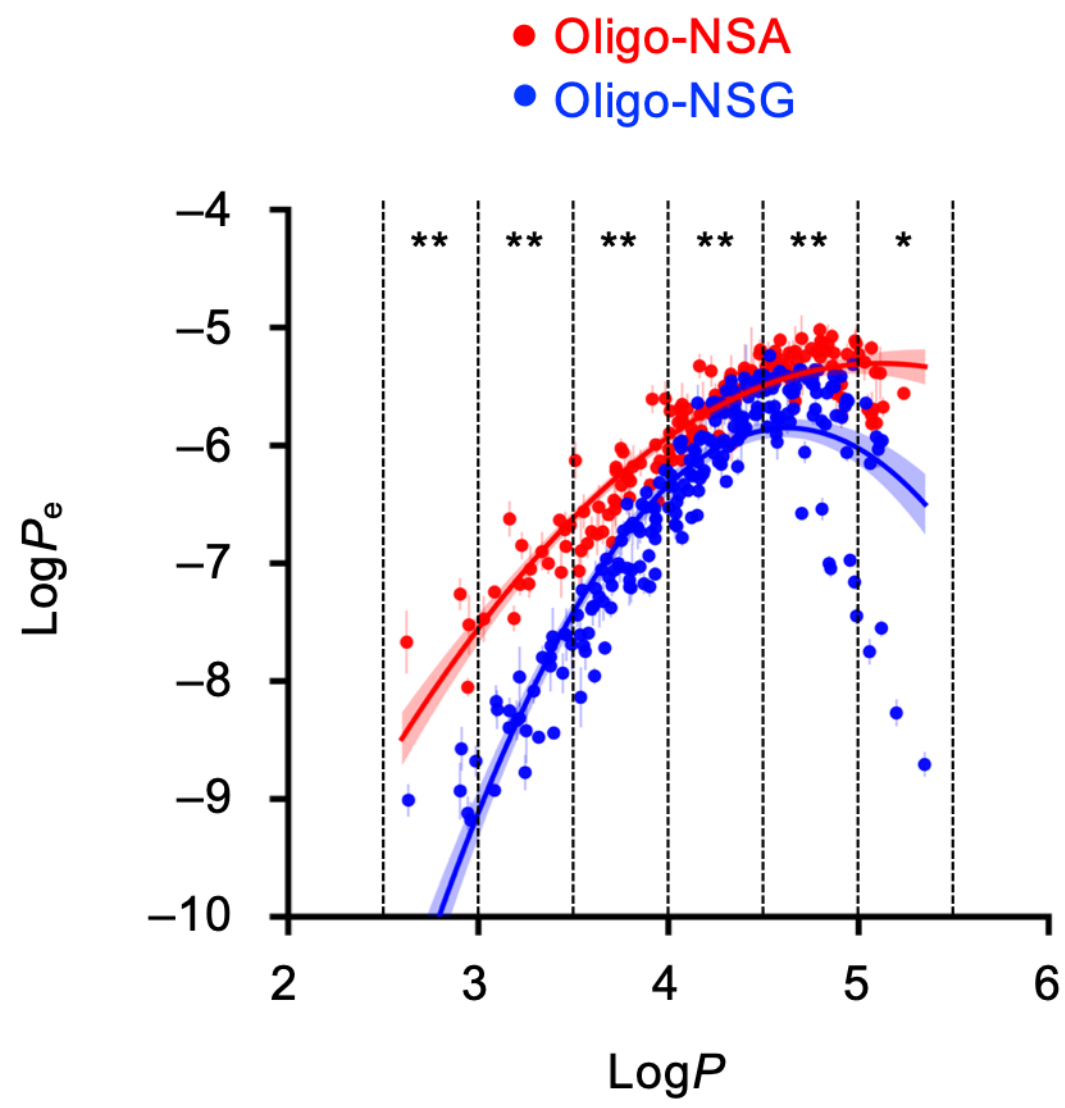

FIGURE S2 | Statistical analysis of the LogP–permeability relationship in the oligo-NSG and oligo-NSA libraries. Relationship between chromatographically determined LogP and PAMPA permeability (LogP_e) for oligo-NSG (blue) and oligo-NSA (red). Solid lines represent separate second-order polynomial regression curves fitted to the two peptide classes, and shaded areas indicate pointwise 95% confidence intervals for the fitted mean responses. For bin-wise statistical analysis, compounds were divided into 0.5-unit LogP bins, and LogP_e values were compared between oligo-NSG and oligo-NSA within each bin using Welch's t-test. Statistical significance is indicated on the graph as $*p < 0.05$ and $**p < 0.01$.

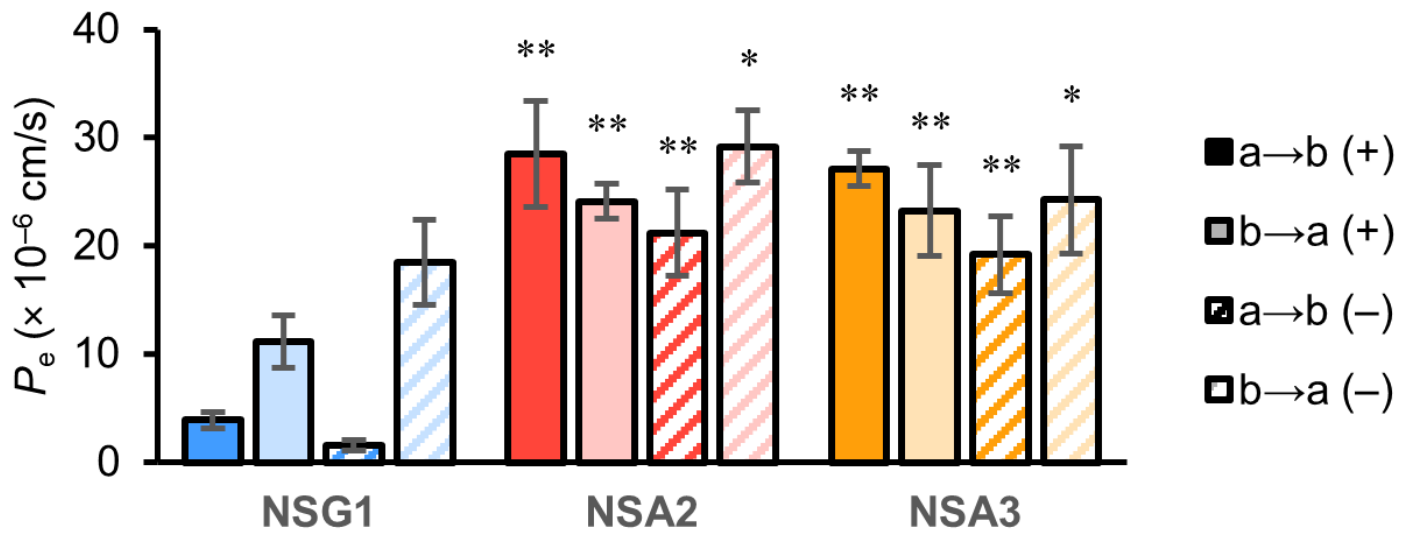

FIGURE S3 | Permeability coefficients determined by the Caco-2 assay. Permeability was measured in the presence (+) or absence (–) of verapamil (100 μM), a transporter inhibitor. P_e values represent the mean ± standard deviation of eight technical replicates for **NSG1** and four technical replicates for **NSA2** and **NSA3**. Statistical significance was evaluated using Tukey's test ($^{**}p < 0.01$, $^{*}p < 0.05$).

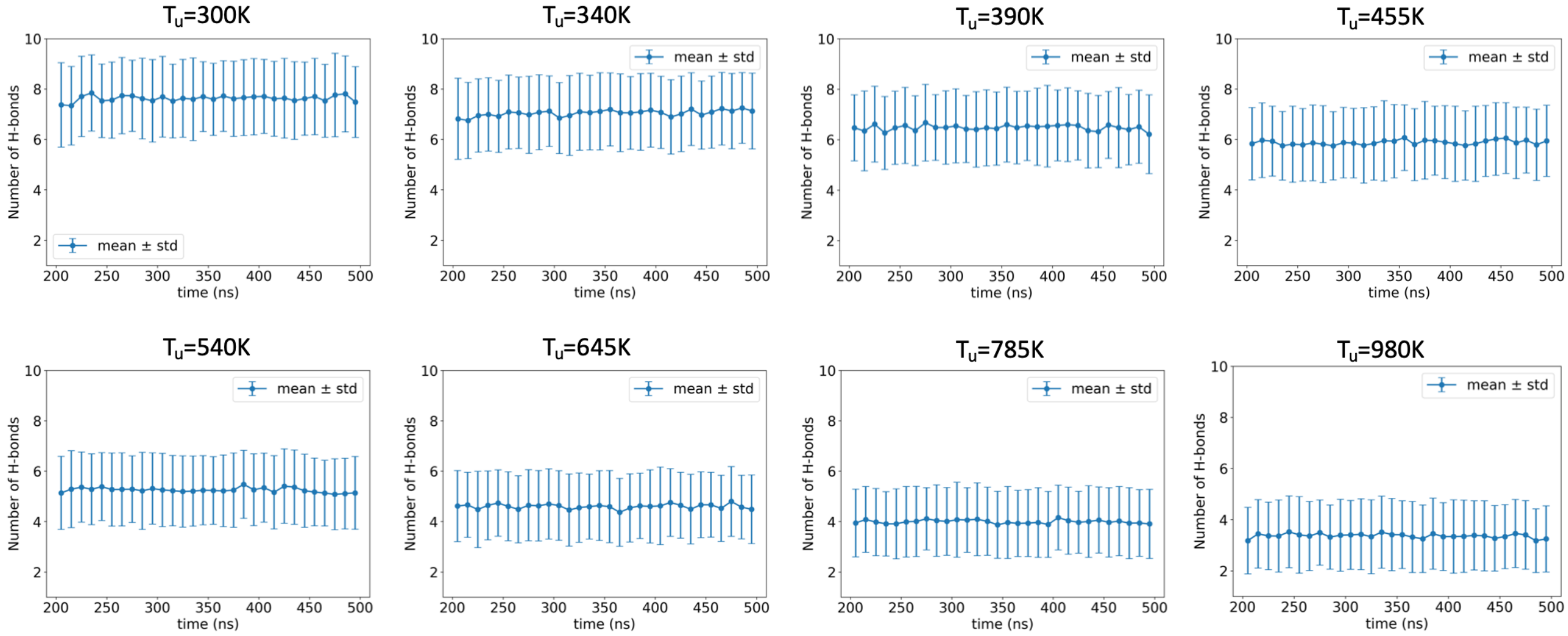

FIGURE S4 | Profiles of the number of peptide–solvent hydrogen bonds as a function of simulation time for the eight REST replicas in the REUS replica located farthest from the membrane in the REST/REUS simulation of **NSA2**. The plotted values represent averages calculated every 10 ns over the 200–500 ns interval used for the analysis, and the error bars indicate the corresponding standard deviations.

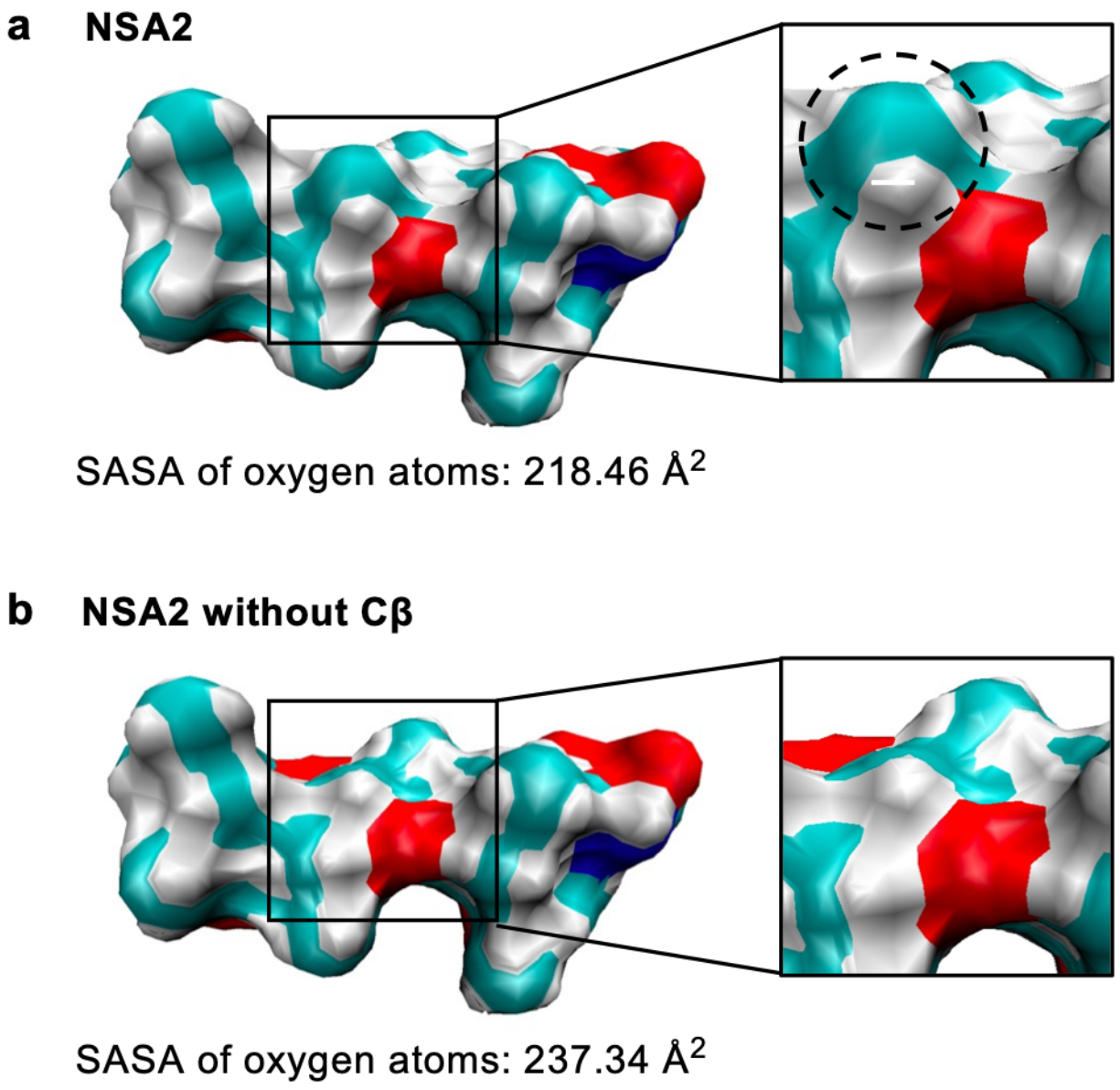

FIGURE S5 | Shielding of oxygen atoms (red) by β-carbons in **NSA2**. Carbon, nitrogen, and hydrogen atoms are shown in cyan, blue, and white, respectively. (a) Representative conformation of **NSA2**. (b) Structure of **NSA2** with β-carbons removed. A representative position from which a β-carbon was removed is closed up on the right and is highlighted with a dotted black circle. The solvent-accessible surface area (SASA) of the neighboring oxygen atom is larger in panel b than in panel a.

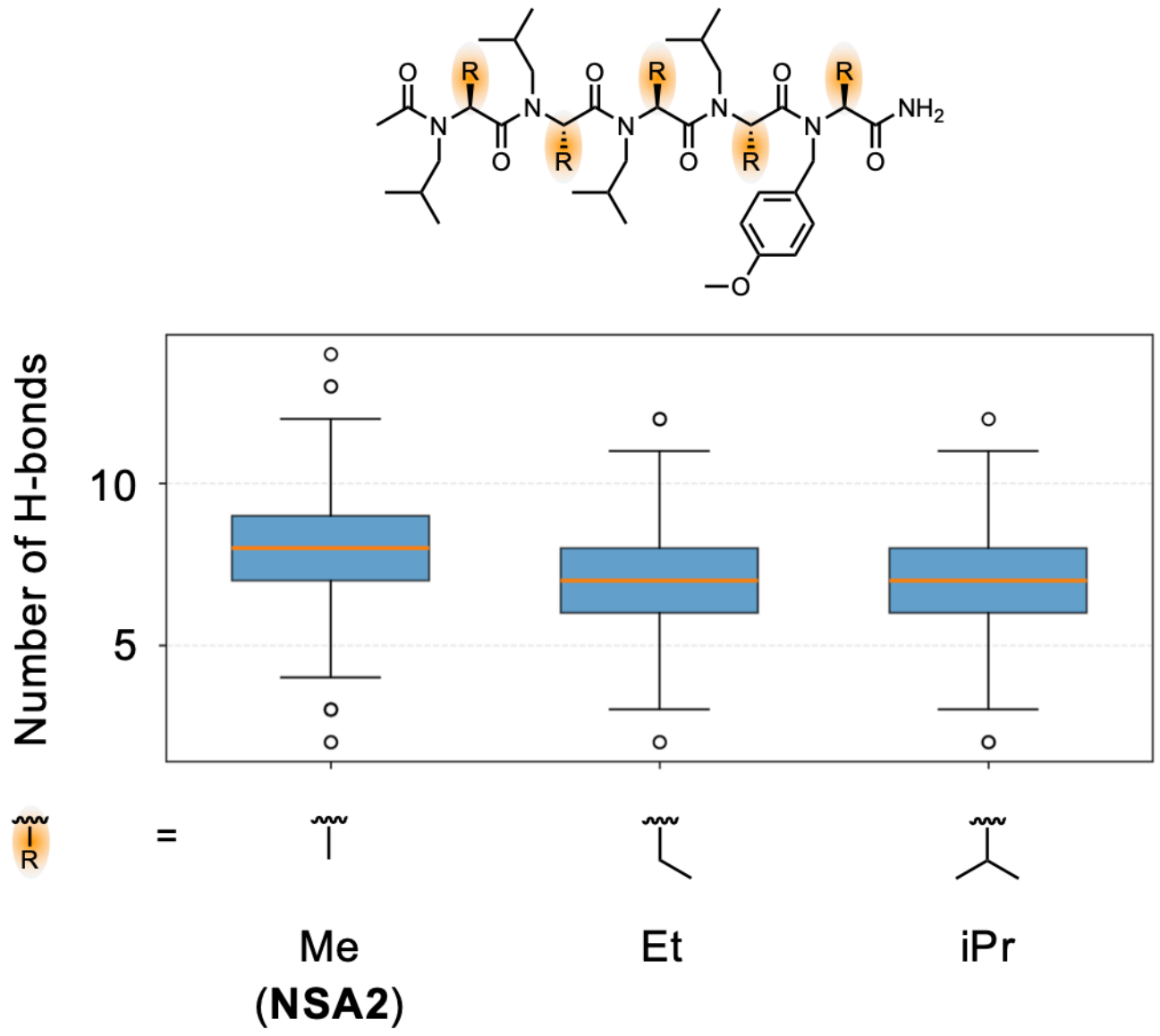

FIGURE S6 | Box plots of the number of solute–solvent hydrogen bonds in the room-temperature replica for **NSA2** and its ethyl- and isopropyl-substituted derivatives in solution. For **NSA2**, the values were taken from the replica located farthest from the membrane in the REST/REUS simulation of the membrane permeation process. For the ethyl- and isopropyl-substituted derivatives, 500-ns REST simulations were performed using eight replicas in a water-filled box, and the values were calculated from the trajectory of the room-temperature replica. The solute temperatures for the replicas were set to 300 K, 340 K, 390 K, 455 K, 540 K, 645 K, 785 K, and 980 K, the same as those used in the REST/REUS simulation.

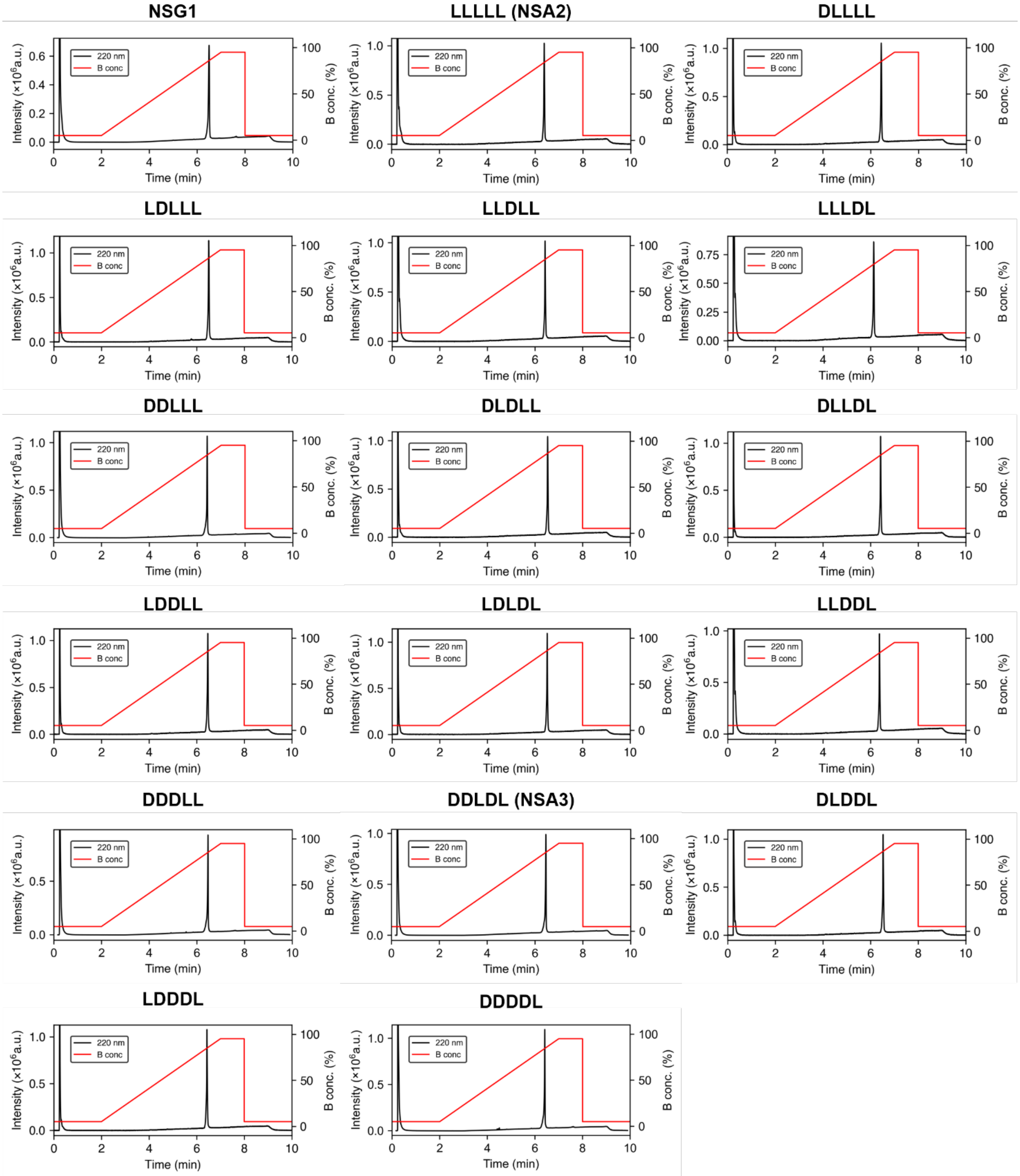

FIGURE S7 | UV chromatograms of purified peptides. Elution was monitored at 220 nm. A binary solvent system consisting of water containing 0.01% formic acid (solvent A) and MeCN containing 0.01% formic acid (solvent B) was used. The gradient profile is indicated by red lines in each chromatogram.

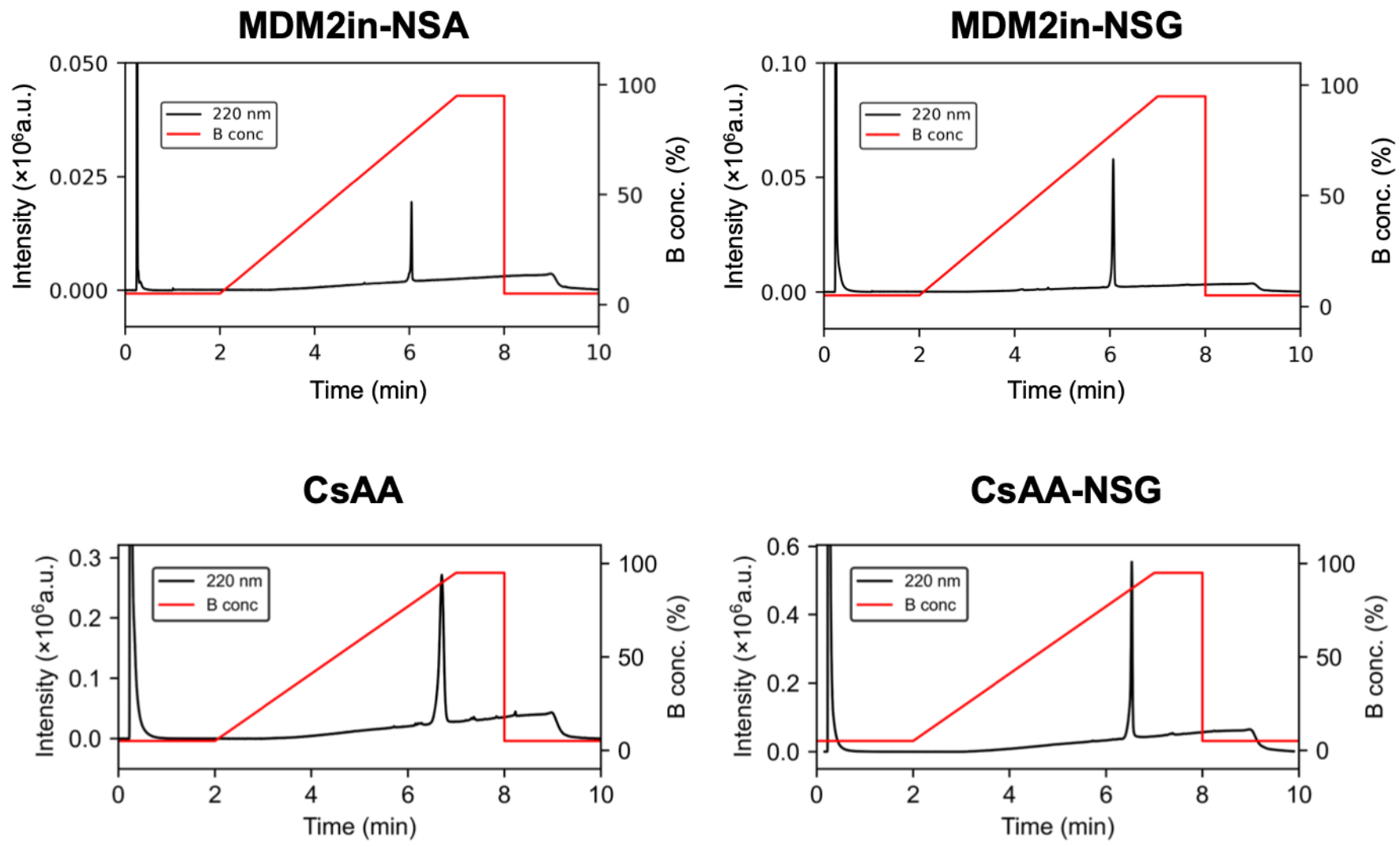

FIGURE S8 | UV chromatograms of the purified **MDM2in-NSA**, **MDM2in-NSG**, **CsAA**, and **CsAA-NSG**. Elution was monitored at 220 nm. A binary solvent system consisting of water containing 0.01% formic acid (solvent A) and MeCN containing 0.01% formic acid (solvent B) was used. The gradient profile is indicated by red lines in each chromatogram.
